# Predictive Wafer-Scale Copper Nanowire Fabrication Using Template-Assisted On-Substrate Electrodeposition

**DOI:** 10.1021/acs.langmuir.5c03780

**Published:** 2025-10-28

**Authors:** Maximilian Vergin, Georg Schöttler, Andreas Waag, Florian Meierhofer

**Affiliations:** Institute of Semiconductor Technology, Nitride Technology Center, 26527 Technische Universität Braunschweig, 38106 Braunschweig, Germany

## Abstract

Precisely engineered metallic nanowire arrays offer a compelling solution for advanced electromechanical interconnects at room temperature, crucial for applications ranging from flexible electronics to 3D integrated circuits. However, their widespread adoption has been hindered by complex and costly fabrication methods. This work reports a streamlined and highly scalable route that overcomes these barriers, enabling the growth of uniform nanowire arrays directly on semiconductor substrates. Our method relies on template-assisted electrodeposition within a simple two-electrode plating chamber. A key aspect of this approach is the use of a melamine foam sponge, which applies uniform mechanical pressure to ensure consistent template-substrate contact and promote homogeneous growth. By combining this reliable synthesis with predictive Monte Carlo modeling of the template morphology, we achieve exceptional control over the final array geometry. Using copper as a model system, our charge-based electrodeposition provides excellent control over nanowire length and yields highly reproducible nanowires with diameters tunable from 100 to 1000 nm and a typical length deviation below ∼20% of the target. The practical utility of this method is validated by demonstrating that these arrays form robust and resilient electromechanical chip-to-chip bonding interfaces with excellent adhesion and conductivity. By providing an accessible and low-cost foundation for producing high-quality nanowires, this work significantly expands their potential for immediate use. This opens up future avenues for developing advanced devices, including high-density vertical interconnects, wearable biosensors, and efficient energy harvesting systems.

## Graphical abstract

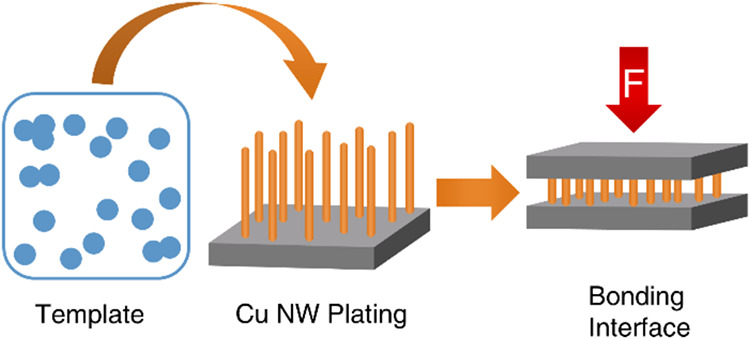

## Introduction

One-dimensional nanostructures, or nanowires (NWs), are distinguished by their submicrometer diameters (typically <1000 nm) and high aspect ratios (often ≫ 1). This pronounced anisotropy imparts unique physical and chemical properties fundamentally different from their bulk counterparts.^1^ For instance, quantum confinement effects within NWs modify their electronic band structures and enable size-tunable optical responses, leading to innovations in nanophotonics such as plasmonic devices and optical waveguides.^2^ Their distinctive mechanical characteristics, offering enhanced flexibility and high specific strength, have been demonstrated to make them ideal for next-generation transparent flexible electronics,^3–5^ high density interconnects,^6–8^ and advanced thermal interface materials.^9–11^ Additionally, the inherently large surface-to-volume ratio of NWs creates a high density of active sites, making them highly interesting for applications in catalysis,^12,13^ sensors,^14–16^ and as advanced electrode materials for batteries.^17–19^ These features also support emerging applications in neural engineering, where NW-based electrodes are being explored as highly localized, biocompatible, and mechanically compliant interfaces for improved brain recording and stimulation.^20^ Collectively, these attributes position NWs as transformative structures.

However, realizing these applications requires precise control over the nanowire material, dimensions, and of particular importance, their collective arrangement and integration with functional substrates and devices. Many conventional top-down or bottom-up synthesis routes often struggle with achieving the desired uniformity, scalability, precise placement, or may require complex postfabrication transfer steps that can compromise wire integrity and device performance.^1,21^ To address these limitations, template-assisted electrodeposition has emerged as a versatile and cost-effective technique for fabricating NW arrays with tunable geometries.^1,22–26^

Commonly used templates for this process include nanoporous anodic aluminum oxide^27^ and ion-track etched membranes.^23–25,28^ This study focuses on ion-track etched membranes, which are fabricated by irradiating polymer films (e.g., polycarbonate or poly­(ethylene terephthalate)) with high-energy heavy ions to create latent damage tracks, followed by selective chemical etching to form cylindrical nanopores of controlled diameter.^29^ Due to the statistical nature of the ion irradiation, the random spatial distribution of the ion-tracks can lead to overlap and the formation of multipore clusters, particularly at higher porosities.^30–32^ Template manufacturers typically report a nominal porosity (φ_nom_), defined as the single-pore area multiplied by the ion-track density. Because overlapping pores reduce the accessible area the nominal porosity overestimates the true open area. Prior studies^30^ addressed this issue by introducing an effective porosity (φ_eff_) that accounts for pore overlap, which has been derived by analytical treatments or small-scale numerical models. Some extend to pairwise overlaps, but closed-form expressions for clusters involving three or more pores are scarce, and broad experimental validation across multiple templates has been limited. A concise summary appears in Supporting Information Table S1. We address this gap by pairing an analytical baseline with Monte Carlo simulations to capture cluster-size distributions, overlap fractions, and shape metrics, and we validate both against an eight-template experimental data set.

Traditionally, template-assisted growth of nanowires has been realized by an on-template electrodeposition strategy, a concept pioneered by Possin in the late 1960s,^33^ where a conductive seed layer is deposited onto the template itself using methods such as sputtering, forming the cathode for the subsequent electrochemical deposition. This concept has been extensively refined over the years and is still frequently considered.^34^ While effective for large-scale production of freestanding nanowires, the on-template approach requires cumbersome and potentially damaging transfer steps when device integration is needed, limiting its suitability for applications that demand direct on-substrate integration, including chip-to-chip bonding.

To overcome these challenges, on-substrate electrodeposition (OSE) grows nanowires directly on the functional substrate, ensuring they are mechanically and electrically integrated from the outset. OSE can be implemented in two ways. In the first, the template is fabricated directly on the target substrate, for example by spin-coating polycarbonate and exposing it to high-energy ion irradiation to form latent tracks that are later etched.^35,36^ This approach is conceptually elegant but requires specialized facilities and multiple process steps. Irradiation can also stress sensitive substrates, which limits applicability. In the second OSE route, a prefabricated template is adhered to the conductive substrate. This approach is gentler, lower cost, and broadly compatible with technologically relevant platforms. Accordingly, we use the adhered-template route. A central requirement for OSE with adhered templates is to achieve and maintain uniform, intimate template–substrate contact over large areas. Nonuniform contact or delamination during electrodeposition causes nonuniform growth, parasitic deposition, and potential device failure. Reported solutions include the use of epoxy resin,^37,38^ liquid film surface tension,^39^ lithography-assisted bonding,^40^ electrostatic adhesion,^9,41^ and mechanical pressure via a melamine foam sponge.^42^ Despite this progress most reports are limited to growth areas of about 1 cm^2^ (see Supporting Information Table S2 for a detailed state-of-the-art summary). Furthermore, while wafer-scale OSE of copper nanowires has been reported, these studies often lack the methodological detail necessary for reproduction, leaving a robust and scalable process unestablished.

In this work, we focus on substrate-integrated copper nanowire arrays grown directly on conductive substrates by on-substrate electrodeposition (OSE). We address key challenges in fabrication scalability, interfacial control, and predictive template modeling. To achieve uniform wafer-scale template-substrate contact, we introduce a robust process combining oxygen plasma surface activation with a melamine foam sponge. We show that nanowire length is precisely prescribed by the passed charge (R^2^ > 0.99) and remains radially homogeneous across full 2-in. wafers. To rationalize and predict template morphology, we pair an analytical effective-porosity baseline with Monte Carlo simulations that quantify pore overlap and cluster-size distributions beyond scalar analytical models. Finally, we demonstrate the application of our OSE fabricated copper nanowires for strong, low-resistance chip-to-chip bonding at room temperature, outperforming thermoplastic adhesives. Together, these results advance nanowire synthesis and provide a robust and scalable foundation for next-generation devices in flexible electronics, catalysis, thermal management, and beyond.

## Experimental Methods

### Nanowire Growth by OSE Process

Substrate-integrated copper NW arrays were fabricated using our on-substrate electrodeposition (OSE) method, the key steps of which are schematically depicted in Figure 1. The figure summarizes the sequence from template mounting and contact, through pulsed electrodeposition of copper, to template removal and exposure of the vertically aligned nanowires.

**Figure 1:**
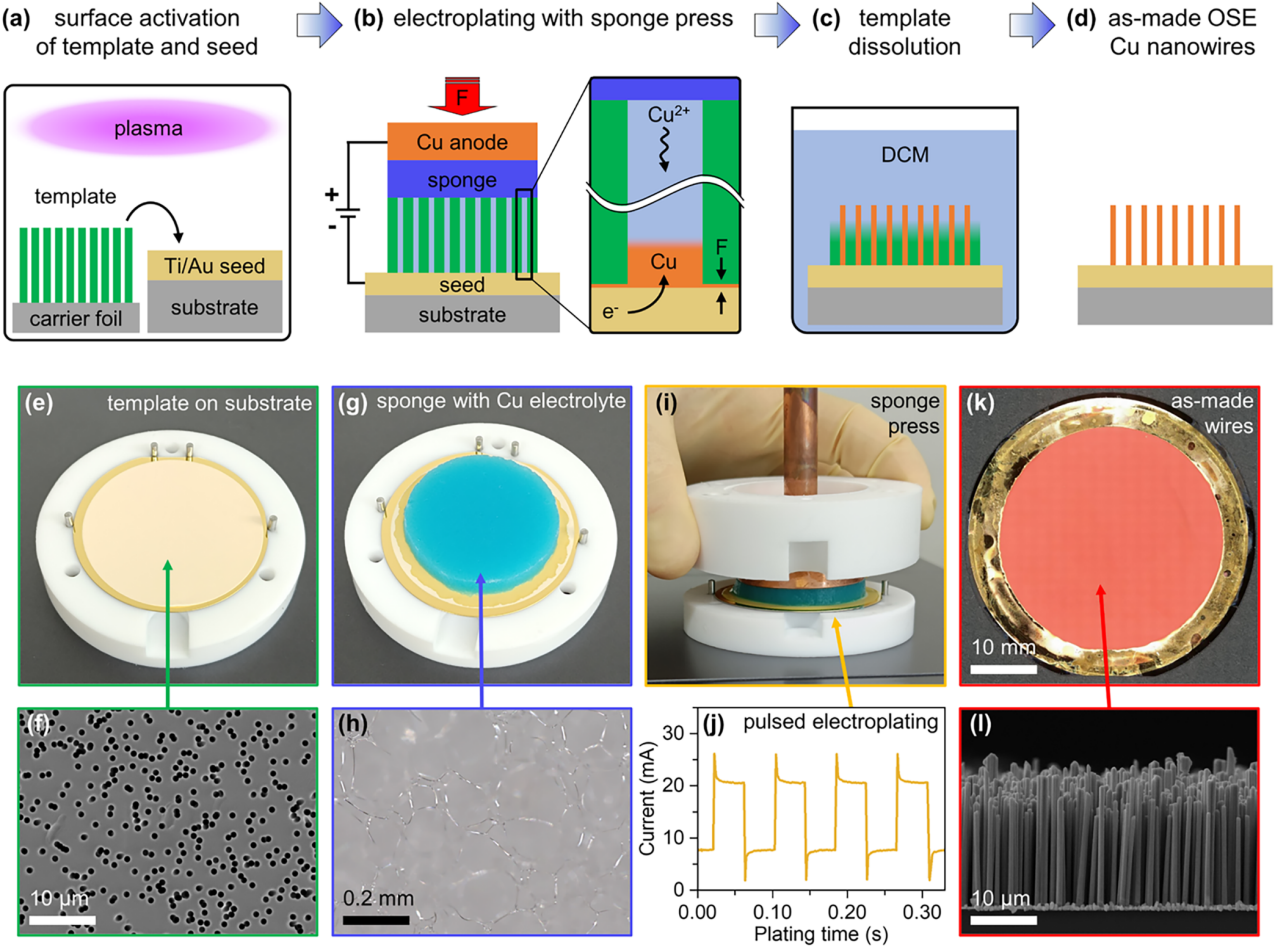
(a–d) Schematic process flow of template-assisted fabrication of copper nanowires with the on-substrate electrodeposition (OSE) process route. (a) The substrate and template surfaces are first activated by an oxygen plasma treatment and then stacked. (b) During electroplating, mechanical force (F) is applied to the copper anode, pressing a melamine foam sponge soaked in copper electrolyte solution onto the substrate. With the template being forced toward the substrate, electroplated copper preferably forms inside the porous template structure. (c) Afterward, the sample is placed in a dichloromethane (DCM) bath to selectively dissolve the polymer template and (d) dried under nitrogen flow. (e) Optical image of the polycarbonate (PC) template on the surface of a 2″ wafer. (f) Top-view SEM close-up of template reveals the nanoporous nature of the template, where the dark circles indicate the pores. (g) Electrolyte-filled melamine foam sponge placed on top of the template. (h) Optical microscope close-up of a dry melamine foam sponge displaying pores that are hierarchically larger compared to the pores of the template. (i) Side-view of the assembled OSE chamber without applying any mechanical pressure to the copper anode. (j) Current–time characteristic of the pulsed electrodeposition. (k) Resulting wafer densely covered with copper nanowires and (l) cross-sectional SEM image highlighting nanowire alignment and uniformity on the wafer surface.

The OSE procedure begins with an oxygen plasma treatment of both the substrate and the nanoporous template (Figure 1a). Next, the core electrochemical cell is assembled. An electrolyte-saturated melamine foam sponge presses the template into uniform contact with the substrate to maintain electrolyte access to all pores during deposition (Figure 1b). After the pulsed electrodeposition of copper is complete, the polycarbonate template is dissolved using dichloromethane (Figure 1c), yielding a substrate-integrated array of vertically aligned copper nanowires (Figure 1d).

To illustrate the process in more detail, Figure 1e shows the initial stacking of the template onto the substrate. A top-down SEM image of a typical template (Figure 1f) reveals the stochastic nature of the pores (dark circles) and highlights instances of pore overlap, which results in multipore cluster formation. Figure 1g displays the melamine foam sponge after electrolyte saturation on top of the template, while Figure 1h highlights the microporous nature of a pristine sponge, respectively. Pressure is applied to the sponge from the top-side via the copper anode (Figure 1i) during the pulsed electrodeposition (Figure 1j). The success of the overall method is confirmed by representative optical microscope and SEM images of the final, dense nanowire arrays (Figure 1k,l).

### Materials

Ion-track etched polycarbonate (PC) templates with poly­(vinylpyrrolidone) (PVP) treatment for improved hydrophilicity were all sourced from the same vendor (it4ip S.A., Belgium) and have specifications as detailed in Table 1. All templates are 47 mm in diameter and have a thickness of approximately 25 μm. Silicon wafers (MicroChemicals) with a (100) orientation were used as substrates. To establish electrical conductivity, wafers were first cleaned using buffered hydrofluoric acid and immediately transferred to a vacuum chamber for e-beam metal evaporation. A 20 nm titanium (Ti) adhesion layer was deposited, followed by a 200 nm gold (Au) conductive layer. The aqueous electrolyte solution comprised 250 g/L copper sulfate pentahydrate (CuSO_4_·5H_2_O, 99% Carl Roth) and 25 g/L sulfuric acid (H_2_SO_4_, 96% MicroChemicals) and was prepared with deionized (DI) water (>18 MOhm). This electrolyte mixture is common in the copper nanowire literature.^43^ The CuSO_4_ acts as the primary source of Cu^2+^ ions, while the H_2_SO_4_ lowers the pH and stabilizes the Cu^2+^ ions in solution, thus enhancing the conductivity and ion mobility of the Cu^2+^ electrolyte, and promoting a more uniform metal deposition.

**Table 1:** Overview of Ion-track Etched Polycarbonate Templates Investigated in This Study^a^

	nominal template parameters				experimental SEM analysis			theoretical analysis	
template	pore diameter, d_p,nom_ (nm)	pore density, ρ_p,nom_ (#/cm^2^)	porosity, φ_nom_ (%)	pore orientation (−)	pore diameter, d_p,SEM_ (nm)	pore density, ρ_p,SEM_ (#/cm^2^)	porosity, φ_SEM_ (%)	analytical eq 7, φ_eff_ (%)	Monte Carlo, φ_MC_ (%)
T1*	100	3.8 × 10^9^	29.85	parallel	106 ± 5	3.55 × 10^9^	25	25.80	25.77
T2*	200	6.0 × 10^8^	18.85	parallel	209 ± 9	6.23 × 10^8^	18	17.18	17.16
T3*	400	2.6 × 10^8^	32.67	parallel	389 ± 11	2.47 × 10^8^	27	27.87	27.87
T4*	400	1.5 × 10^8^	18.85	multiangle	387 ± 11	1.66 × 10^8^	19	17.18	17.18
T5*	1000	2.2 × 10^7^	17.28	multiangle	949 ± 21	2.36 × 10^7^	17	15.87	15.87
T6	200	1.0 × 10^9^	31.42	parallel	196 ± 5	9.41 × 10^8^	26	26.96	26.93
T7	200	1.3 × 10^9^	40.84	parallel	187 ± 19	1.27 × 10^9^	34	33.53	33.49
T8	100	5.1 × 10^9^	40.06	parallel	104 ± 3	4.55 × 10^9^	32	33.01	32.96

a Manufacturer-specified nominal pore characteristics are listed alongside experimental SEM-determined pore diameters, densities, and porosities. Effective porosities are derived analytically (eq 7) and by Monte Carlo simulation. The standard deviation of the porosity measurements is approximately 1% for all measurements. Templates indicated with “*” were used for copper nanowire growth via our on-substrate electrodeposition (OSE) method.

### Deposition Setup

Fabrication of copper nanowires on 2″ substrates was realized by using a self-designed plating chamber composing of a baseplate and a cylindrical center part both made from poly­(tetrafluorethylene) (PTFE). The anode was made from oxygen-free high conductive (OFHC) copper and had a cylindrical shape fitting into the PTFE center part. The anode was cleaned with H_2_SO_4_ (1 mol/L) immediately before beginning the plating procedure. The melamine foam sponge pressing onto the templates had a thickness of 0.5 cm (BASF, Basotect). It was cut into circles with the same diameter as the Cu anode and immersed in the electrolyte solution to ensure full saturation before use in the plating chamber. Mechanical force was applied from the top onto the Cu anode using a manually operated z-stage equipped with an integrated force gauge to measure the applied force.

### Electrodeposition Protocol

Prior electrodeposition, wafers and PC templates underwent RF low pressure oxygen plasma treatment (TePla, 100-E). The wafers were treated at 150 W for 60 s to remove organic residues to boost adhesion and enhance hydrophilicity to promote wetting. The template surface intended for contact with the wafer was plasma-treated at 150 W for 30 s. After plasma treatment, the substrate and template were directly utilized for nanowire fabrication. The plating chamber was then assembled according to the following steps: (1) The baseplate was submerged in deionized (DI) water, and the wafer was placed onto it. (2) The template was submerged, then positioned with its plasma-treated side facing the wafer. (3) The core chamber was mounted onto the baseplate and secured with screws. (4) Trapped DI water was gradually replaced with the electrolyte, and the system was allowed to rest for 30 min to ensure complete wetting. (5) The electrolyte soaked melamine foam sponge was then placed atop the wafer-template stack, followed by the anode. (6) Electrical connections were established using crocodile clips and a mechanical force of approximately 50 kg (456 kPa) was applied to the anode. This applied pressure was selected based on preliminary trials indicating optimal template-substrate contact, crucial for uniform and complete pore filling. Lower pressures risk insufficient contact, leading to uneven deposition and parasitic deposition between template and substrate, while excessive pressures can overly compress the melamine foam sponge, squeezing out electrolyte and reducing electrolyte access to the pores. Thus, the chosen pressure effectively balances reliable contact with sufficient electrolyte pore access, consistently resulting in reproducible and uniform nanowire growth. (7) Pulsed electrodeposition was initiated using a source measuring unit (Keithley, SMU 2614B), with alternating potentials of 0.1 V for 40 ms and 0.05 V for 40 ms. As dissolved metal ions diffuse more freely inside the channels during the off-cycle time, the pulsed operation mode can be beneficial for template-assisted electroplating of nanowires or other high aspect ratio structures.^44–47^ Electrode potentials, pulse durations, and deposition parameters presented here were empirically chosen based on initial tests that consistently produced uniform, high-quality nanowires for this combination of copper electrolyte and substrate material. The SMU is controlled by a custom Python software script designed for precise length control of the OSE-made nanowires. To convert a desired nanowire length into a charge-based stopping criterion, we apply Faraday’s law. As Faraday’s law of electrolysis states, the mass gain (*m*) relates to the passed charge (Q_plating_) according to

1 $$m=\frac{\eta M_{\mathrm{Cu}}}{\mathrm{zF}}Q_{\mathrm{plating}}$$

Here, the current efficiency (η) is an empirically determined parameter, *M*_Cu_ = 63.546 g/mol is the molar mass of copper, *z* is the electron number (*z* = 2 for Cu^2+^ → Cu), and *F* = 96,485 C/mol is Faraday’s constant. The mass of the deposited copper can also be expressed as the total volume of the nanowire array (*V*_NW_) times the density of copper (ρ_Cu_ = 8.96 g/cm^3^). Furthermore, the array volume is a function of the substrate area (*A*_sub_), the template porosity (φ), and the average nanowire length (*L*_NW_), which gives

2 $$m=\rho_{\mathrm{Cu}}V_{\mathrm{NW}}=\rho_{\mathrm{Cu}}\varphi A_{\mathrm{sub}}L_{\mathrm{NW}}$$

Combining eqs 1 and 2 then yields an expression for the predicted nanowire length (*L*_pred_) as a function of passed charge

3 $$L_{\mathrm{pred}}\left(Q\right)=\frac{m}{\rho_{\mathrm{Cu}}\varphi A_{\mathrm{sub}}}=\frac{\eta M_{\mathrm{Cu}}}{\mathrm{zF}\rho_{\mathrm{Cu}}\varphi A_{\mathrm{sub}}}Q_{\mathrm{plating}}$$

To fabricate nanowires of a desired length, the Python script inverts eq 3 to calculate the required target charge (*Q*_target_). During deposition, the software integrates the current in real-time and automatically terminates the process once *Q*_target_ is reached. For all experiments, the substrate plating area was *A*_sub_ = 11.94 cm^2^, and the porosity (φ) was set to the manufacturer’s nominal porosity (φ_nom_) for the chosen template.

### Postgrowth Treatment

After plating, the chamber is disassembled and the substrate is rinsed multiple times with DI water to remove residual electrolyte. To dissolve the PC template, the substrate is then immersed in dichloromethane (DCM, 99.8%, Carl Roth) for at least 1 h with periodic solvent exchanges. Afterward the wafer was again placed in DI water to remove the residual DCM before drying the wafer in a pure nitrogen flow.

### Characterization of Templates and OSE Nanowires

Scanning Electron Microscopy (SEM, TESCAN MIRA3) was used to precisely measure pore density and pore diameter of the porous PC templates. To mitigate charging effects in insulating polymer material, the PC templates were coated with a 5 nm titanium (Ti) layer by electron beam (e-beam) evaporation prior to the SEM investigations. The high directionality of the e-beam evaporation ensures uniform coating without clogging the pores, which is crucial for accurate pore characterization.

For the OSE copper nanowires, an optical microscope (Keyence, VHX7000) was used as a first measure of the homogeneity of the nanowire growth process. Gravimetric analysis of samples before and after the nanowire growth was performed using a precision scale (Mettler Toledo, XPR205DR) to calculate mass gain due to the metal deposition and the current efficiency. SEM analysis was then utilized to investigate the geometrical characteristics of OSE-grown copper nanowires as described in the Results section. Over 1000 nanowires were counted across multiple SEM images to determine cluster statistics. To measure nanowire length, the wafers were cleaved perpendicular to the wafer flat and then again perpendicular to this edge, resulting in four quarter circles. SEM images of the nanowires were then taken along these straight edges at 20 distinct sites per wafer. For nanowire length and diameter measurements several hundred nanowires were measured across these 20 sites. The software ImageJ was used to extract measurements from the SEM images.

### Monte Carlo Simulation

To investigate pore clustering beyond the scope of analytical models, Monte Carlo simulations were developed in Python. For each template specified in Table 1, the simulation followed three key steps, with further details and a visual workflow provided in the Supporting Information Figure S1. First, pore centers were placed within a defined two-dimensional area using a uniform random number generator. Second, a Density-Based Spatial Clustering of Applications with Noise (DBSCAN) algorithm was used to identify clusters of overlapping pores. A cluster was defined as any group of two or more pores where the distance between adjacent centers was less than the mean pore diameter. Finally, to accurately calculate the area of each cluster, the identified pore coordinates were converted to a pixel-based representation, and a flood-fill algorithm was applied to determine the total area, which correctly accounts for any enclosed voids within a cluster. We use these simulations to reproduce and quantify the clustering observed in SEM and to interpret its implications for array morphology. The validated framework provides a path toward template-specific prediction of OSE nanowire morphology.

### Electromechanical Characterization of NW Array Bond

To evaluate the electromechanical properties of the NW array interconnects, a bonded sample was prepared and tested. Two chips (approximate area: 1 cm × 1 cm) from a wafer grown using the T4 template (mean nanowire length: 18.5 μm) were pressed together with a force of approximately ∼10 kg to form the initial bond. Electrical characterization was performed before and after mechanical testing using a Keithley 4200A-SCS Parameter Analyzer. To facilitate measurements from a single side of the bonded assembly, copper tape (CMC Klebetechnik) with conductive adhesive was carefully attached to the exposed conductive layer of each chip substrate. For mechanical pull-testing, the bonded sample was mounted into a custom-built tensiometer. Two cylindrical metal rods (5 cm long, 8 mm diameter) were attached to the backside of each silicon chip using Crystalbond 509, a thermoplastic adhesive that was melted to create a strong bond and then cooled to solidify. One rod was attached to a stationary fixture connected to a digital force gauge (Erichsen, model 709), while the other was attached to a moveable stage. A tensile force was applied by moving the stage at a constant rate, and the force was recorded until failure occurred (see Supporting Information Video S1). Following the mechanical test, the failed interfaces were inspected via optical microscope.

## Results and Discussion

### Characterization of Pores in Ion-Track Etched Templates

Precise control over NW array geometry, and thus device performance, relies on an accurate understanding of the structural parameters of ion-track etched membranes used as templates. Deviations from manufacturer specifications, particularly regarding pore diameter, pore density, and pore distribution, can significantly affect the nanowire properties and functionality. We quantify pore diameter, pore density, and porosity by SEM and compare them with manufacturer data and model predictions. To interpret template porosity it is essential to distinguish nominal porosity (φ_nom_) and effective porosity (φ_eff_). This distinction and the underlying analytical model for effective porosity were established by Riedel and Spohr.^30^ Template manufacturers typically report the nominal porosity (φ_nom_), which is calculated by multiplying the single-pore area with the ion-track density

4 $$\varphi_{\mathrm{nom}}=\frac{N\pi {d_{p}}^{2}}{4A}$$

Here, the pore diameter (*d*_p_), the number of ion tracks (*N*) per template area (*A*) are known parameters. However, as Riedel and Spohr demonstrated for pores placed with a uniform random spatial distribution, higher pore densities inevitably lead to pore overlap, reducing the true open area.^30^ The probability that a randomly chosen point remains uncovered by *N* independently placed pores can be approximated by

5 $$p_{\mathrm{uncovered}}\approx \exp\left(-\frac{N\pi {d_{p}}^{2}}{4A}\right)$$

Consequently, the effective porosity, representing the true fraction of open area, is given by

6 $$\varphi_{\mathrm{eff}}=1-p_{\mathrm{uncovered}}\approx 1-\exp\left(-\frac{N\pi {d_{p}}^{2}}{4A}\right)$$

By inserting eq 4 into [Disp-formula eq6] the effective porosity can be expressed in terms of the nominal porosity

7 $$\varphi_{\mathrm{eff}}\approx 1-\exp\left(-\varphi_{\mathrm{nom}}\right)\leq \varphi_{\mathrm{nom}}$$

This framework shows that the effective porosity is always below the nominal value, with the gap increasing at higher porosities as overlap becomes more prevalent. Consequently, nominal porosity calculations overestimate the open area. We characterized eight ion-track templates (T1–T8) using the workflow described in Supporting Information Figure S1. Table 1 compiles the manufacturer specifications together with SEM-derived diameters, densities, and porosities, and reports effective porosity from eq 7 and from Monte Carlo simulation. Five templates (T1–T5, marked with an asterisk) were subsequently used for OSE growth of copper nanowire arrays and set the target charges in our growth series.

SEM analysis in Figure 2a–e visually confirms the pore clustering phenomenon in our templates. To gain deeper insight, we developed a Monte Carlo simulation to model this complex morphology. Using the template parameters in Table 1, the simulations generate spatial pore arrangements (Figure 2f–j) that display a striking visual resemblance to the experimental SEM images. This correspondence indicates that the simulations correctly capture the statistical nature of pore placement and overlap.

**Figure 2:**
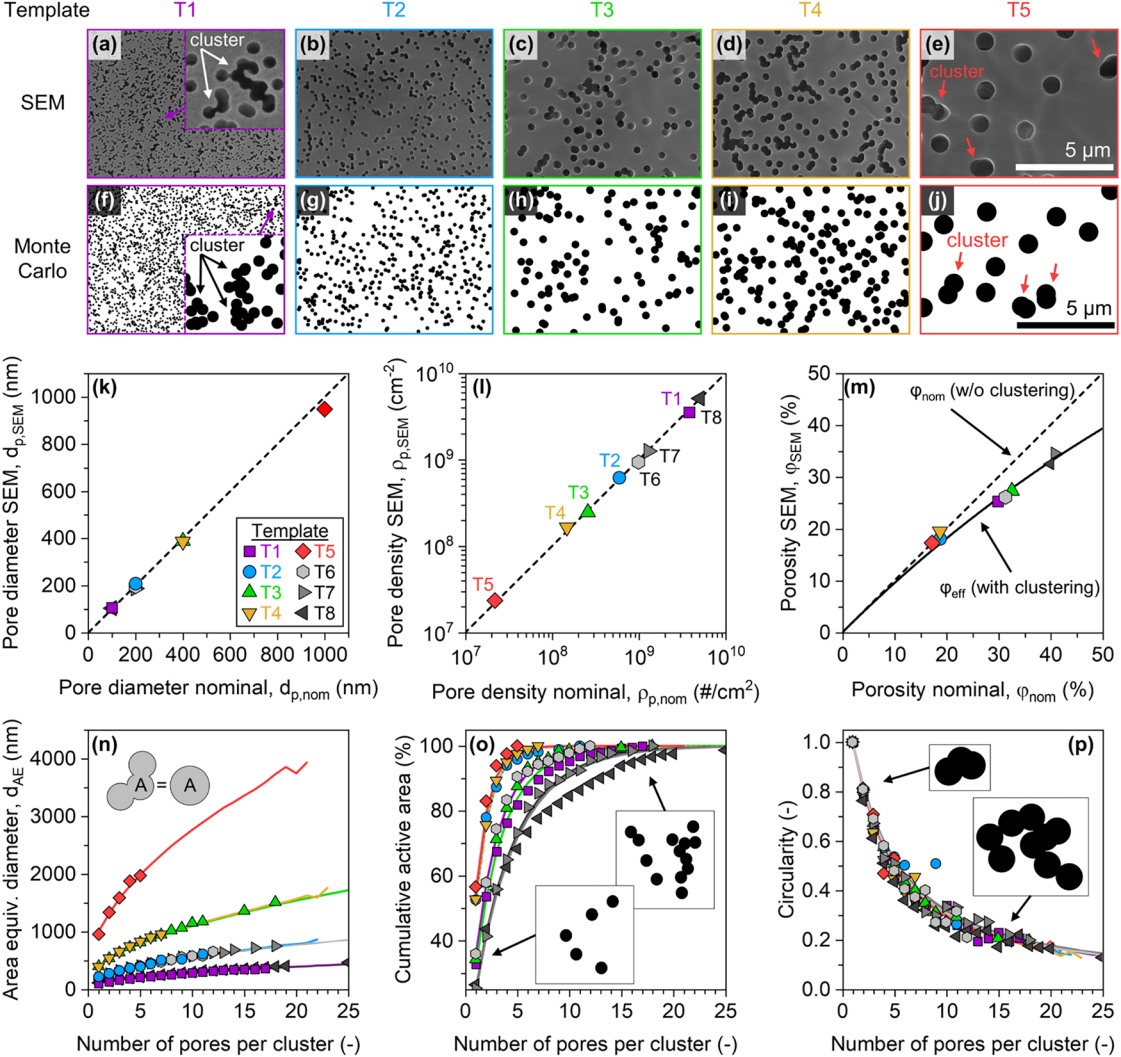
Statistical characterization of ion-track etched membranes. (a–e) Representative scanning electron microscopy (SEM) images showing typical pore morphologies of the investigated templates. (f–j) Corresponding pore morphologies generated by Monte Carlo simulations, closely reproducing experimental observations. (k) Comparison between manufacturer-specified and experimentally measured pore diameters, demonstrating close agreement. (l) Comparison of manufacturer-specified versus experimentally determined pore densities, confirming the reliability of manufacturer specifications. (m) Comparison of the nominal porosity, the analytical effective porosity, and the SEM measured porosity. (n) Area-equivalent diameter comparison between experimentally observed and simulated pore clusters, highlighting the accuracy of the simulations. (o) Cumulative pore area fraction as a function of cluster size, emphasizing the importance of multipore clusters. (p) Pore circularity analysis versus cluster size comparing experimental results to Monte Carlo simulations, validating the predictive capability of our model. Lines indicate simulation data for the template parameters.

With the template morphology characterized, we next performed a quantitative analysis, beginning with individual pore features. The measured pore diameters (Figure 2k) and pore densities (Figure 2l) were found to be in good agreement with the manufacturer’s nominal values. However, a significant discrepancy emerges when considering the overall porosity, as illustrated in Figure 2m. The plot shows that at higher nominal porosities (φ_nom_), the manufacturer’s calculation based on eq 4 increasingly diverges from the analytical model for effective porosity (φ_eff_, eq 7). Crucially, our experimentally measured porosity values (φ_SEM_, detailed for each template in Table 1) closely follow the trend predicted by the φ_eff_ model, thereby affirming its underlying assumptions and highlighting the importance of accounting for pore overlap and clustering. For example, for T3 the nominal porosity is φ_nom_ = 32.67%, while the SEM-measured porosity is φ_SEM_ = 27%, a deviation of +5.67 percentage points. The overlap-corrected analytical model predicts an effective porosity φ_eff_ = 27.87%, and the Monte Carlo estimate is φ_MC_ = 27.87%, both in good agreement with SEM. Across all eight templates, the deviation φ_nom_ – φ_SEM_ ranges from −0.15 to +8.06 percentage points. Comparing φ_SEM_ with φ_eff_ and φ_MC_ across all templates yields root-mean-square errors of 1.05 percentage points and 1.04 percentage points, respectively, which are comparable to our measurement uncertainty of approximately 1 percentage point. Analytical and Monte Carlo porosities are nearly identical because both compute the same scalar quantity under random placement.

When nanowires grow within clustered pores they merge into multipore features with two main consequences. First, the accessible surface area decreases relative to an array of individual nanowires, potentially diminishing performance in applications like catalysis and sensing. Second, the mechanical behavior of the NW arrays changes. Individual copper nanowires exhibit distinct size-dependent properties, such as a transition in deformation behavior around 100 nm in diameter.^48^ Clustering shifts the effective dimension upward, which can modify mechanical and electrical characteristics, and, in turn, affect device reliability and performance.

### Monte Carlo Simulation of Pore Clustering

While the effective porosity (φ_eff_, eq 7) captures the open area, it does not describe cluster geometry. We therefore use Monte Carlo simulations to gain deeper insights into the specific geometry and distribution of pore clusters. The simulations allow for a more detailed comparison with experimental template features than analytical models alone. Details of the Monte Carlo simulation methodology, including pore generation, cluster identification using DBSCAN, and area calculation via flood-fill algorithms, are provided in Supporting Information Figure S2.

To assess the accuracy of the model, we compared several geometric metrics. First, we calculated the area-equivalent cluster diameter (d_AE_), which is given by the diameter of a circle having the same area as the pore cluster (*A*_cluster_)­

8 $$\left(d_{\mathrm{AE}}=\sqrt{4A_{\mathrm{cluster}}/\pi }\right)$$

Figure 2n plots the area-equivalent cluster diameters for both the experimental SEM data and Monte Carlo simulations. The close correspondence between the two data sets confirms that the simulations accurately replicate the experimentally observed trend, highlighting that clusters are monotonously larger than their single-pore counterpart.

Furthermore, Figure 2o illustrates the cumulative area covered by clusters of increasing size, providing critical insight into the pore overlap. Both experimental data and simulations show that at higher nominal porosities, the template’s open area becomes dominated by multipore clusters rather than isolated single pores. This effect is particularly present in template T8 (φ_nom_ ≈ 40%), where single pores account for less than 30% of the total open area. Hence, most nanostructures grown using this template will form within these multipore clusters, possessing effective dimensions considerably larger than the nominal single pore diameter, as highlighted in Figure 2n.

Finally, we examined the circularity of the clusters as a metric for their shape. The circularity of a cluster with area (*A*_cluster_) and perimeter (*P*_cluster_) is given by

9 $$\mathrm{circularity}=\frac{4\pi A_{\mathrm{cluster}}}{P_{\mathrm{cluster}}^{2}}$$

The circularity approaches unity for a perfect circle and decreases for more complex or elongated shapes. Figure 2p examines the circularity of clusters over cluster size, affirming that both experimental and simulated data follow the same trend. The consistently decreasing circularity for larger clusters indicates that the clusters tend to grow elongated as they incorporate more pores. This elongation is further illustrated by representative ellipses and measured clusters in Support Information Figure S2.

These simulation results closely match our experimental measurements across cluster size, area distribution and circularity, and they show the value of numerical modeling beyond scalar analytical estimates for realistic template morphology. The Monte Carlo statistics quantify the extent and geometry of multipore clusters that we observe in experiment and they help interpret array properties. Larger cluster fractions and sizes reduce the share of isolated pores, which lowers accessible surface area for catalysis or sensing, while increasing effective feature size and local stiffness. For our target application of bonding, moderate clustering may aid mechanical interlocking, whereas excessive clustering can reduce conformability. In this study we use Monte Carlo primarily to validate and interpret the observed morphology. Using these statistics to guide template selection and process parameters will be the focus of future work.

### On-Substrate Electrodeposition (OSE) of Copper Nanowire Arrays

Ideally, the morphology of the nanoporous template is directly transferred into the morphology of the copper NW array. Since the accuracy of this replication is a critical factor for applications like chip-to-chip interconnects, the structural properties of the copper NW arrays fabricated via the OSE method were systematically analyzed. Figure 3a–j provides a comprehensive overview of the fabricated nanowire samples with additional images provided in the Support Information Figures S3–S11. The direct comparison of the NW geometry and the templates structure reveals that our on-substrate electrodeposition (OSE) approach faithfully replicates the template morphology. The fabricated NW arrays show excellent agreement with those of the associated templates. Specifically, the nanowire diameters (d_NW,SEM_) match the template pore diameters (Figure 3k), and the area-equivalent diameters of nanowire clusters (d_NW‑AE,SEM_) scale consistently with those of the corresponding pore clusters (Figure 3l,m). Furthermore, the measured nanowire density aligns closely with the nominal pore density (Figure 3n), indicating that issues such as pore blockage or incomplete nucleation are negligible in this process.

**Figure 3:**
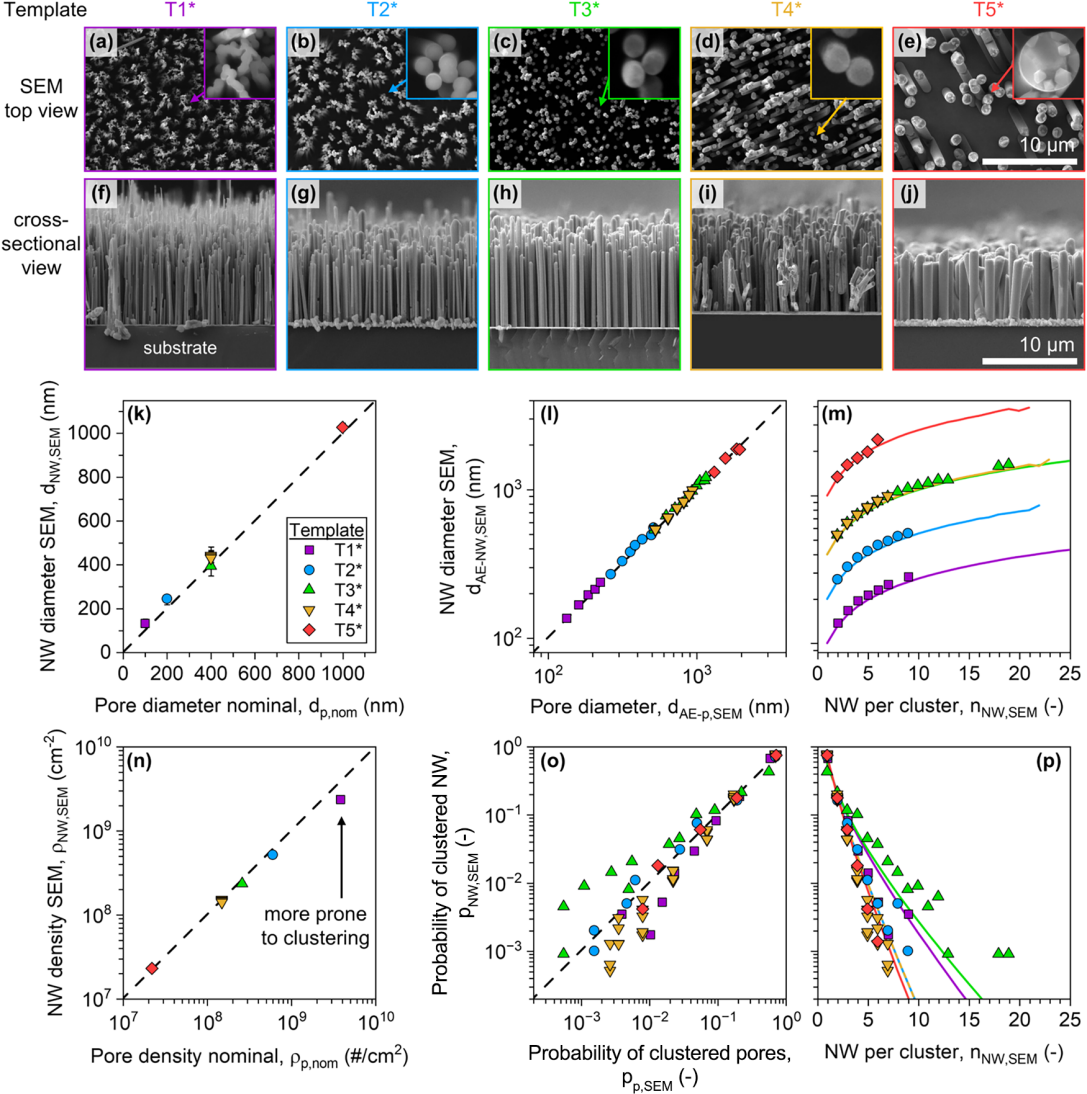
Statistical characterization of grown nanowires using our on-substrate electrodeposition (OSE) method. (a–e) Representative scanning electron microscopy (SEM) images showing the top view of the nanowires, which is used to assess the cluster formation and the nanowire density. (f–j) Corresponding SEM cross-section images are employed to measure the length at different sites. (k) Comparison between nanowire diameter and measured pore diameters, demonstrating close agreement. (l, m) Measurement of the area equivalent diameter for the clustered nanowires and the comparison with clusters measured in the templates. (n) Comparison of the measured density in the nanowires and the measured density in the corresponding templates. (o, p) Determination of the cluster occurrence probabilities and comparison to the occurrence probabilities in the template. Lines indicate simulation data for the respective templates.

Deviations between copper nanowire morphology and pore morphology are quantitatively described by the pore-filling efficiency (η_fill_), which is calculated by comparing the measured nanowire density (ρ_NW,SEM_) to the nominal template pore density (ρ_nom_)­

10 $$\eta_{\mathrm{fill}}=\rho_{\mathrm{NW},\mathrm{SEM}}{/\rho }_{\mathrm{nom}}$$

In our case, the pore-filling efficiency consistently exceeds 90% for most templates. The lowest pore filling efficiency observed was roughly 63% for template T1. For this high-aspect-ratio sample, SEM analysis reveals significant postgrowth contraction and bunching of nanowires (Figure 3a), likely caused by capillary forces during solvent evaporation, a well-known challenge in drying dense nanostructures.^7^ These forces can physically detach nanowires from the substrate or contract them into dense bundles that prevent accurate characterization, both of which can lead to an underestimation of nanowire density. These effects can be reduced, with added process complexity, by supercritical drying,^7^ solvent exchange to a lower-surface-tension drying solvent,^49^ or slower solvent evaporation in a controlled atmosphere. The high replication accuracy demonstrated across the other templates (T2–T5) confirms the overall robustness of our on-substrate electrodeposition method. Crucially, this high degree of replication achieved in our OSE process allows the Monte Carlo model of the initial template to serve as predictive model for the final nanowire array structure. As shown in Figure 3o,p, the probability distribution of nanowire cluster sizes not only matches that of the template pores but also confirms excellent agreement with the predictions from our simulations. The model also correctly captures the influence of template parameters, clearly distinguishing between templates of low and high effective porosity, where higher porosities lead to a significantly larger probability of forming larger clusters (Figure 3p).

This validated link between initial template design, simulation, and the final experimental nanowire array provides a powerful framework. It moves beyond simple trial-and-error fabrication to enable the rational design of template geometries, allowing for the engineering of nanowire arrays with predictable and optimized functional properties for applications such as advanced interconnects, catalysis, and sensing.

### Tailoring Nanowire Length and Uniformity on full Wafers

For transferring the nanowire process into a robust industrial process queue, controlling the nanowire length, diameter and uniformity is of utmost importance. As governed by Faraday’s law of electrolysis, nanowire length (*L*_NW_) is directly proportional to the total charge passed during deposition (see eq 1–3). This linear relationship allows for predictable tuning of the final NW array geometry. Therefore, chronoamperometry provides critical real-time insight into the evolution of nanowires within the template material, and hence can be used to control the growth process *in situ*. The recorded chronoamperograms for all of our samples exhibit features characteristic of template-confined growth (Supporting Information Figure S12). After the initial, brief current spike associated with double-layer charging at the electrolyte-substrate interface, we find that the current rapidly decays to a steady level of 10–20 mA. This rapid decay, without a prolonged period at a higher intermediate current, is a key indicator for immediate pore confined growth and is comparable to chronoamperograms observed for on-template electrodeposition systems, where intimate contact is guaranteed.^50^ This confirms that our simple sponge-pressed on-substrate electrodeposition approach achieves immediate and stable template-substrate contact, which is the foundation for precise control over nanowire length and uniformity.

Our experimental results confirm this predictable linear scaling. The measured nanowire length exhibits a strong linear correlation with the deposition charge (*R* ^2^ > 0.99), as shown in the length series in Figure 4a–e and summarized in Figure 4f. For example, a deposition charge of 123 As resulted in a NW array with a mean length of 17.68 ± 2.63 μm, representing the longest nanowires grown on this linear trend.

**Figure 4:**
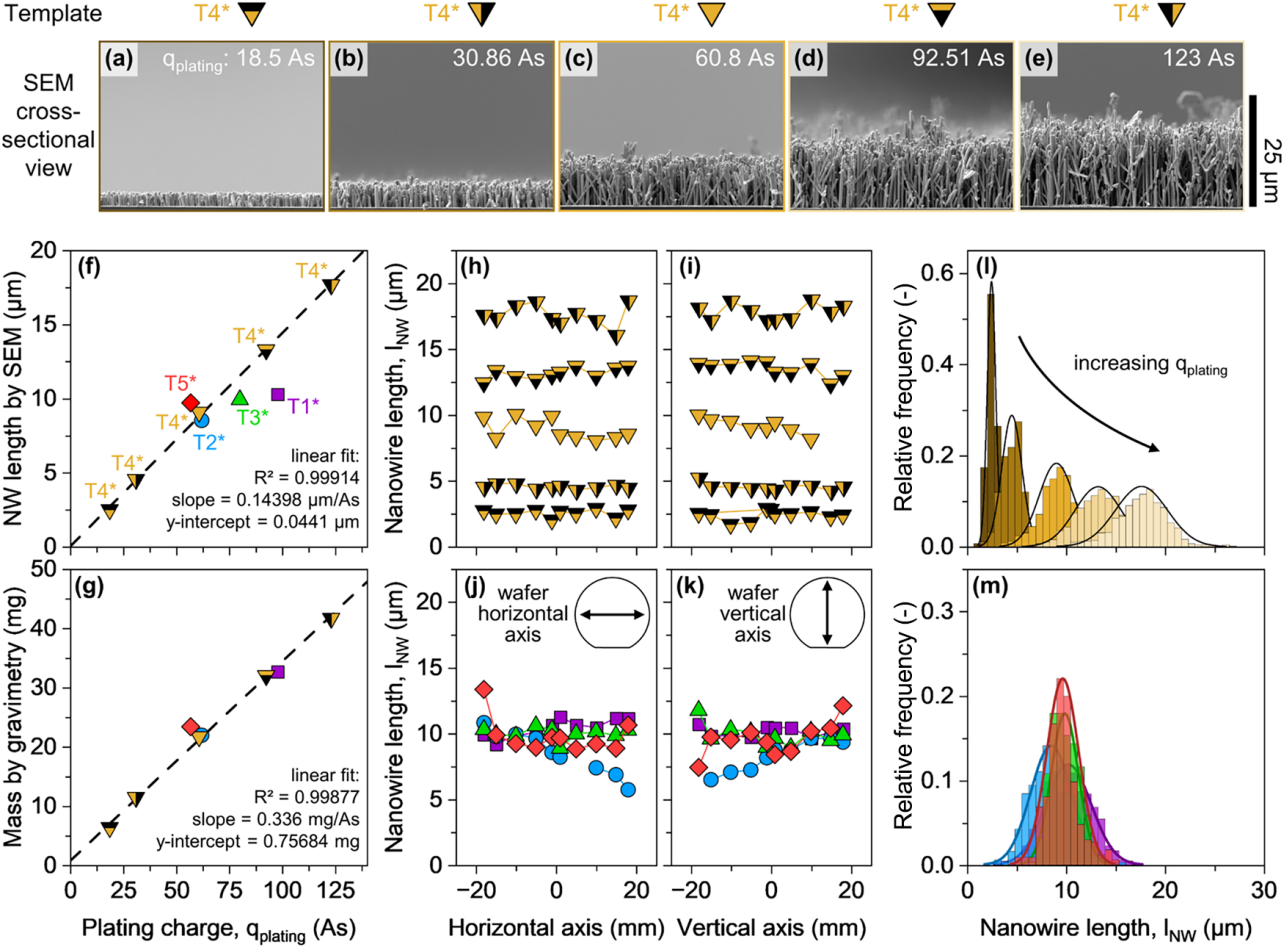
Statistical length characterization of grown nanowires. (a–e) Representative scanning electron microscopy (SEM) images corresponding to cross-section images of nanowires at different heights for the same template. (f) Comparison between the measured nanowire length and the theoretically expected length using Faraday’s law. (g) Measurement of the gained mass of the samples compared to the flown charge and expected mass gain from the charge. (h-i) Comparison of the measured length spanning along the main axes of the wafer for the different lengths of the same template T4. (j, k) Length uniformity along the main axes for all other samples (T1, T2, T3, and T5). (l) Distribution of the nanowire length for the samples shown in (h, i). (m) Distribution of the length for the samples shown in (j, k).

This high degree of control is further supported by mass deposition measurements. The measured mass gain during electrodeposition also scales linearly with the passed charge (Figure 4g), providing independent validation of the process. These results confirm that the process operates with a consistently high and stable Faradaic efficiency, calculated to be 99% ± 2%. This near-complete conversion indicates that the charge is almost exclusively used for the electrochemical reduction of copper ions. Combined with the morphological evidence from SEM analysis (cf. Figure 3a–e), this high efficiency verifies that the deposited copper predominantly forms the intended nanowire structures. The SEM images verify that parasitic copper deposition on top of or underneath the template is negligible, and that the process is dominated by growth within the template pores.

Furthermore, the on-substrate electrodeposition method provides excellent wafer-level length uniformity. For various target lengths using template T4, the local mean nanowire length deviates, on average, by less than 10% from the wafer-level mean (Figure 4h,i). These findings confirm homogeneous nanowire deposition over large areas. This level of control is not limited to a single template geometry. By adjusting the deposition charge in accordance with Faraday’s law to account for different template parameters, distinct templates (i.e., T1, T2, T3, T5 in Figure 4j,k) can be used to fabricate NW arrays of the same target length with the predicted mass gain. Overall, for all sample nanowire samples, the length distribution histograms given in Figure 4l,m exhibit a narrow spread with a typical standard deviation of roughly 20% relative to the mean length.

While alternative methods like template overgrowth followed by a peel-away process have been reported to yield higher uniformity,^9^ they introduce significant drawbacks. Such methods limit scalability, restrict length tunability (length is fixed by template thickness), and may suffer from incomplete nanowire array transfer, critically undermining their suitability for scalable device fabrication. Other approaches using thermal gradients to improve uniformity significantly complicate the experimental setup and reduce deposition rates, undermining process simplicity and throughput.^46,51^ In contrast, our OSE method strikes a compelling balance between high uniformity, process simplicity, and length tunability, making it well-suited for scalable and flexible nanowire array fabrication. Further improvements, such as chemical-mechanical planarization after growth, could be employed if higher uniformity is required, albeit at the cost of an additional processing step.

### Room-Temperature Electrical Chip-to-Chip Bonding with OSE Nanowire Arrays

To demonstrate the functional utility of the OSE-fabricated nanowire arrays, we evaluate the arrays as a room-temperature electrical chip-to-chip interconnect. The targeted application domain is microelectronic packaging and heterogeneous integration where low-temperature bonding avoids thermal budgets associated with solder reflow or thermocompression adhesives. Two chips from a single wafer (template T4, mean nanowire length 17.68 μm) are assembled at room temperature under a nominal bonding pressure of approximately 1 MPa to form a bonded interface (Figure 5a). The electromechanical integrity of this bond is then assessed using the electrical measurement scheme shown in Figure 5b. SEM imaging of the interface reveals that the high-density nanowires create an intertwining structure, similar to hook-and-loop fastener, forming a robust mechanical and electrical connection at room temperature (Figure 5c). First, current–voltage (*I*–*V*) measurements confirm the formation of a conductive network across the bonded interface (Figure 5d, prepull *I*–*V* curve). The mechanical strength of the nanowire-bonded chip-to-chip interface is measured by a custom pull-test apparatus applying axial load to the bond, as illustrated in the schematic of Figure 5e.

**Figure 5:**
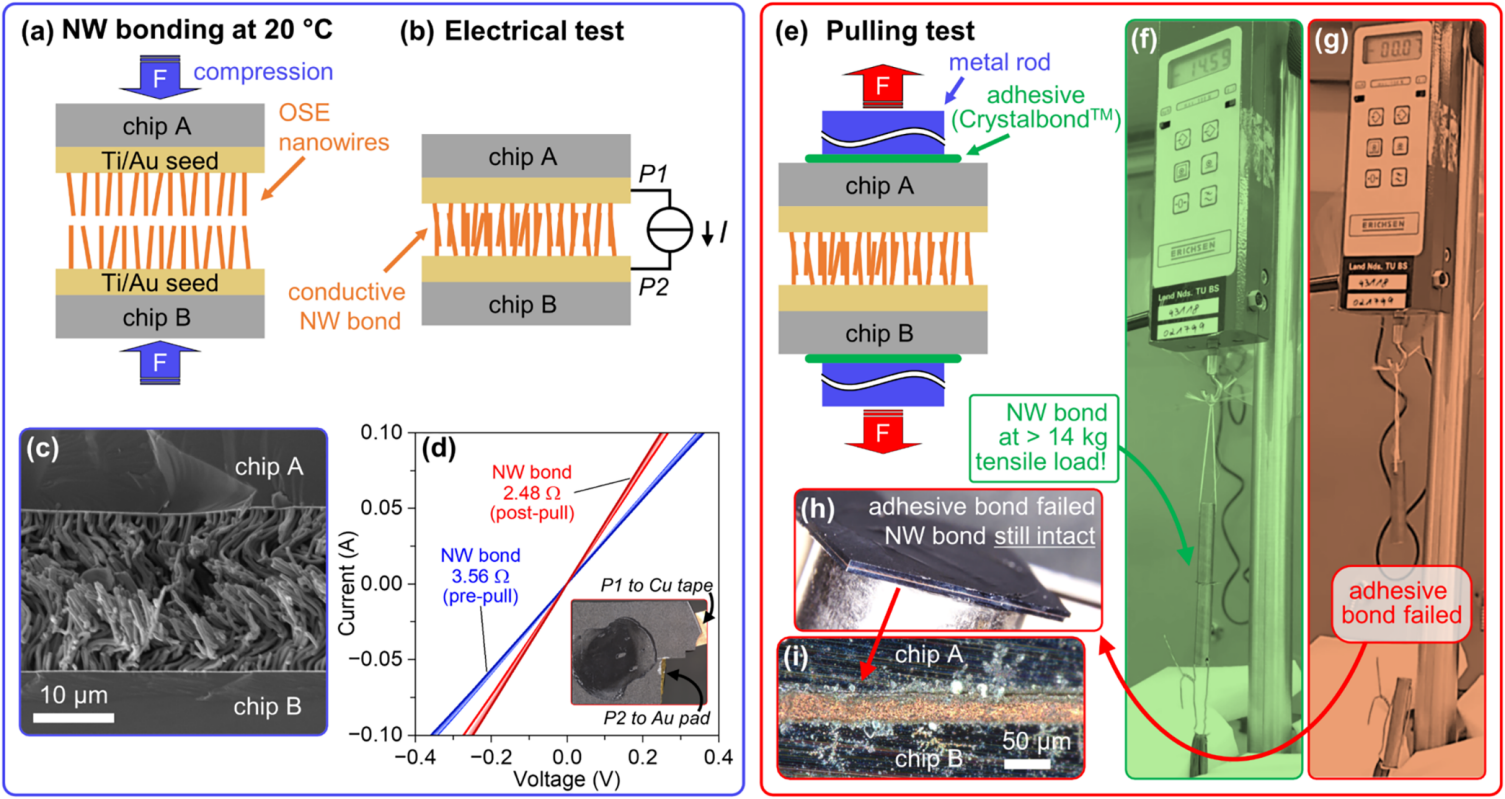
Demonstration of NW array based electromechanical bonding. (a) Schematic of two NW array coated chips pressed together to form a bonded interface. (b) Schematic of probe setup used for electrical characterization of the bonded interface. (c) Cross-sectional SEM image of the intertwined nanowire interface, illustrating the Velcro-like interlocking mechanism. (d) Current–voltage (*I*–*V*) characteristics of the bonded interface measured before (pre-pull) and after (post-pull) the mechanical pull-test. (e) Schematic of the pull-test setup, showing the bonded sample attached to a force gauge and mechanical stage via metal rods and adhesive. (f–i) Series of photographs images showing the sequence of the pull-test, highlighting the failure of the Crystalbond adhesive rather than the NW-NW bond interface.

During the pull-test, we found that the NW array bond proves to be remarkably strong and remains intact without any signs of failure (Figure 5f–i). Mechanical failure occurred in the thermoplastic adhesive (Crystalbond) that was used to attach the sample to the test setup (see Supporting Information Video S1). The recorded force in the moment of failure is ∼142 N (∼14.5 kg tensile load), which, applied over the 8 mm diameter contact area, corresponds to a failure stress of approximately 2.8 MPa at the adhesive-wafer interface. This provides a lower bound of ≥2.8 MPa on the NW-NW interfacial strength, set by failure in the external adhesive. Crucially, after being subjected to these significant mechanical loads, the bonded interface remains electrically conductive, exhibiting *I*–*V* characteristics comparable to the pretest measurements (Figure 5d, postpull *I*–*V* curve).

This successful demonstration confirms that the nanowire interconnect is not only strong but also remarkably resilient, maintaining its electrical integrity even when stressed to the point of external system failure. Forming strong bonds at room temperature is a critical advantage for heterogeneous integration of temperature-sensitive materials. The precise control of nanowire geometry, density, length, and uniformity achieved here underpins the observed performance. These results establish feasibility for room-temperature electrical interconnects and highlight the potential of OSE for reliable, large-area components in low-temperature wafer bonding, flexible electronics, and thermal interface materials.

## Conclusion

In conclusion, we have established a comprehensive methodology for the fabrication, characterization, and predictive modeling of substrate-integrated copper nanowire arrays. Our on-substrate electrodeposition (OSE) approach, using a simple two-electrode setup and a melamine foam sponge for uniform template contact, provides a robust, scalable, and reproducible route to synthesizing metal nanowire arrays with precise control over key structural parameters, including diameter, density, clustering, and length. A central achievement of this work is bridging the gap between template specifications and the final, as-grown nanowire morphology. Our rigorous study demonstrated that while nominal template specifications can be misleading at higher porosities, the true template structure is accurately captured by our Monte Carlo simulations. The excellent agreement between simulations and experiments, coupled with the accurate replication of the template, confirms that the geometry of these nanowire ensembles is both highly predictable and controllable. This predictive capability fundamentally changes the approach to nanowire fabrication, moving it from a trial-and-error process to one of rational design. By demonstrating strong, resilient, room-temperature electromechanical chip-to-chip bonding, we confirmed the practical utility of our OSE copper nanowires.

To place our OSE route in context with other transfer-free approaches, we highlight three key differences. First, relative to direct on-substrate ion-track templating,^35^ our adhered-template OSE avoids the specialized irradiation step, reduces substrate risk, retains electroplating flexibility, and enables immediate electrical and mechanical integration. Second, top-down lithography plus metal etch or electroplating fill can produce ordered arrays, but 100 nm diameters at high aspect ratios demand advanced tools and unfavorable throughput and cost.^2^ Our method achieves tall, submicrometer features over wafer areas with simple hardware and low process complexity. Third, metal coating of nonmetal pillars avoids metal etching but leaves a nonconductive core that sets a minimum effective diameter and can distort surface topography.^21^ Selective core removal, for example sacrificial or etch-back schemes, is possible in principle yet adds steps and can leave fragile shells. Our arrays are solid metal by construction and are electrically and mechanically integrated to the substrate from the start. Adhered track-etch polycarbonate templates are inexpensive and can reach very high structure densities and aspect ratios. The trade-off is stochastic clustering and lack of long-range order. However, in this work we demonstrate that this randomness in template morphology, and therefore array morphology, is predictable with Monte Carlo simulations, enabling rational design at scale.

Beyond interconnects, the same OSE framework is promising for catalysis and biosensing because it couples wafer-scale reproducibility with tunable geometry. Control over length, diameter, density, and clustering sets geometric surface area and transport pathways, while solid-metal, directly attached wires minimize series resistance and remain compatible with wet functionalization. In principle the process is not limited to copper and can be applied to other electroplatable metals^52^ and alloys,^34^ and combined with postgrowth transformations to oxides or sulfides, which broadens the accessible chemistries.

## Supplementary Material





## Notes

Additional experimental details; literature summaries (Tables S1–S2); template-pore characterization workflow and Monte Carlo procedure; circularity analysis of pore clusters; SEM cross sections of nanowire length; chronoamperograms for potentiostatic deposition (Figures S1–S12) (PDF)
Pull-test demonstration for chip-to-chip bonding (Video S1) (MP4)
