# Supplementary material for: Predictive Wafer-Scale Copper Nanowire Fabrication Using Template-Assisted On-Substrate Electrodeposition
Source: Langmuir. 2025 Oct 28;41(44):29627–39. doi: 10.1021/acs.langmuir.5c03780 (PMC12613796; doi:10.1021/acs.langmuir.5c03780)
Supplement: Supplementary file 1 [file la5c03780_si_001.pdf]

Supporting Information

Predictive Wafer-Scale Copper Nanowire Fabrication using Template-Assisted On-Substrate Electrodeposition

Maximilian Vergin[1], Georg Schöttler[1], Andreas Waag[1], Florian Meierhofer[1]**

[1] Institute of Semiconductor Technology, Nitride Technology Center, Technische Universität Braunschweig, 38106 Braunschweig, Germany

* Corresponding authors:
m.vergin@tu-braunschweig.de, f.meierhofer@tu-braunschweig.de

Supporting Information

Table S1: Literature review of pore distribution statistics of ion-track etched membranes.

Table S2: Literature summary of on-substrate electrodeposition (OSE) using ion-track etched membranes.

Figure S1: Workflow for experimental template characterization and Monte Carlo simulation.

Figure S2: Circularity of experimental and reconstructed pore clusters.

Figure S3-11: SEM cross sectional analysis to reveal length of OSE copper nanowires at various radial distances.

Figure S12: In potentiostatic template-assisted electrodeposition, chronoamperograms (left colored axes) help monitoring the evolution of copper metal nanowires.

Video S1: Demonstration of pull-test for nanowire chip-to-chip bonding.

Table S1: Literature review of pore distribution statistics of ion-track etched membranes. Abbreviations: Analytical calculation (AC), Monte Carlo Simulation (MC).

Reference (year, author, affiliation)	Model Type	Pore Shape	Material	Porosity Range (%)	Max. Cluster Size (pores)	Key Findings	Validation	Limitations
Ref. [1] (1979, Riedel and Spohr, GSI Helmholtz Centre)	AC, MC	Quadratic	Mica	Analytical: ≤ 20<br>Monte Carlo: ≤ 140	≤ 7	Introduced concept of effective porosity due to overlap; linear correlation between pore area and cluster size.	—	Simulation size limited to 200,000 pores,<br>analytical model valid only for low porosities (< 20%), assumes uniform pore shape,<br>2D simulation
Ref. [2] (1980, Riedel and Spohr, GSI Helmholtz Centre)	MC	Rhomboidal	Mica	4.7 – 42.5	≤ 7	Good agreement between MC simulation and experimental SEM of etched mica.	Experimental (SEM of etched mica)	Limited to specific material (mica),<br>assumes uniform pore shape
Ref. [3] (1981, Riedel and Spohr, GSI Helmholtz Centre)	AC, MC	Square	None	≤ 30	≤ 11	Provided analytical and numerical methods to correct observed counts due to overlapping at high event densities	Compared analytical model against MC simulation results	No area calculations,<br>model limited to clusters of ≤11 pores,<br>assumes uniform pore shape
Ref. [4] (1994, Kim, University of New South Wales)	Image Analysis Statistics (IAS)	Circular	PC	1 – 20	≤ 4	Analyzed pore shape variations and porosity relationships (IAS)	—	Only empirical (no simulation),<br>low cluster sizes,<br>assumes uniform pore shape
Ref. [5] (1995, Calvo, University of Valladolid)	Image Analysis Statistics (IAS)	Circular	PC	4 – 16	≤ 5	Detailed analytical treatment of pore size distribution obtained by IAS	—	Limited to small clusters ≤5,<br>only empirical (no simulation),<br>analytical fitting of measurement data limits insight,<br>assumes uniform pore shape
Ref. [6] (2001, Shorin, Institute of Physics and Power Engineering Russia)	AC, MC	Circular, Quadratic	-	≤ 50	≤ 3	Analytical model for cluster (≤ 3) probabilities	—	No validation,<br>limited to small clusters ≤3,<br>assumes uniform pore shape
Ref. [7] (2003, Shorin, Institute of Physics and Power Engineering Russia)	AC, MC	Circular	-	≤ 30	≤ 4	Angular dependence on pore formation	—	Focuses mostly on the angular spread,<br>simulation size limited to 12,000 pores per run, assumes uniform pore shape
Ref. [8] (2022, Sawada, TIARA Japan)	MC	Circular	-	Sim: 4.5 - 90	≤ 11	Modeled effective porosity and visualized cluster formation at various porosities	—	10x10 μm² sim area,<br>does not mention encircled areas,<br>assumes uniform pore shape,<br>single plane simulation
Ref. [9] (2024, Jianqiang, Tsinghua University)	MC	Circular	PC	Sim: ≤ 60,<br>Exp: 11.7, 15.9	≤ 5	Simulated effective porosity, and visualized cluster formation and area	Experimental (2 PC membranes)	Area calculation neglects encircled voids,<br>200x200 μm² sim area,<br>Large pore diameter (≥2.6 μm) limits cluster complexity,<br>assumes uniform pore shape,<br>2D simulation
This work	AC, MC	Circular	PC	Sim: No Limit,<br>Exp: 17.28 – 40.84	Sim: No Limit,<br>Exp: ≤ 25 observed	Analytical porosity calculation, simulated characteristics of clusters	Systematic validation against 8 commercial PC membranes (17 - 41% porosity)	Assumes uniform pore shape,<br>cluster area is a tradeoff between accuracy and computational power,<br>only one manufacturer,<br>single plane simulation

Table S2: Literature summary of on-substrate electrodeposition (OSE) using ion-track etched membranes. Abbreviations: Galvanostatic Deposition (GSD, plating at constant current), potentiostatic deposition (PSD, plating at constant voltage), pulsed electrodeposition (PED), copper sulfate (CS), copper sulfate pentahydrate (CSH), sulfuric acid (SA). $ Value calculated from data in the cited reference. # Claim made without explicit supporting evidence in the publication.

**Reference (year, author, affiliation)**	**Material**	**Electrolyte**	**No. electrodes, plating mode**	**Growth area (cm^2)**	**Pore diameter (µm)**	**Pore density (1/cm^2)**	**Porosity**	**Wire length (µm)**	**Pore Orient**
Ref. [10] (2004, Vila, UCLouvain)	Cobalt	CoSO4 (0.8M), H3BO3 (0.4 M)	3, PSD (-)	-	0.02	1E7	<0.1$	2.1	Parallel
Ref. [11] (2007, Yoon, University of Arkansas)	Gold<br>Polypyrrole<br>Platinum<br>Ruthenium	Technic TechniGold 25ES<br>Pyrrole (-), NaClO4 (-)<br>Technic platinum-S<br>Technic ruthenium-U	3, PSD (- 0.65 V)<br>3, PSD (0.5 V)<br>3, PSD (- 1.5 V)<br>3, PSD (- 1.5 V)	< 1	0.01<br>0.2<br>1	4E8<br>-<br>2E7	<0.1$<br>-<br>15.7$	2	Multiangle
Ref. [12] (2011, Gambirasi, CNR IENI Milan)	Copper<br>Nickel	CS (0.88 M), SA (0.55 M)<br>NiSo4.6H20(1.126 M), Nicl26h20 (0.185 M), H3BO3 (0.485 M)	2, PSD (- 0.4 V)<br>2, PSD (- 0.7 V)	0.78	1<br>0.4<br>0.2	2E7<br>1E8<br>3E8	15.71$<br>12.57$<br>9.42$	8	Multiangle
Ref. [13] (2013, Cui, Nagoya Unviersity)	Copper	CSH (0.4 M), SA (5 mM$)	-, GSD (5 mA)	0.03	0.15	-	-	-	Multiangle
Ref. [14] (2013, Greiner, TU Darmstadt)	Gold<br>Copper<br>Nickel	Metakem Gold-SF<br>CSH (1 M), SA (0.2 M)<br>Ni(SO3NH2)2 (1.1 M), H3BO3 (0.56 M)	3, PSD (- 0.5 V)<br>3, PED (100 ms / - 0.2 V; 200 ms / 0 V)<br>3, PED (10 ms / -0.55 V; 20 ms / 0 V)	78#	0.1<br>0.2<br>0.36<br>1<br>1.6	4E8<br>3E8<br>1E8<br>2E7<br>4.3E6	3.14$<br>9.42$<br>10.18$<br>15.71$<br>8.65$	100	Parallel, Multiangle
Ref. [15] (2013, Wang, Nagoya University)	Copper	CSH (0.4 M), SA (5 mM$)	-, GSD (3 mA)	0.64$	0.15	-	-	20	Multiangle
Ref. [16] (2014, Motoyama, Stanford University)	Nickel	NiSO4 (1 M), H3BO3 (0.62 M)	3, PSD (- 0.9 V)	< 0.01					
Ref. [17] (2015, Barako, Stanford University)	Copper	CS (0.6 M), SA (30 mM)	3, PSD (- 0.12 V)	1	0.2<br>0.4<br>1.0	2.4E8<br>1.3E8<br>2E7	7.54$<br>16.34$<br>15.71$	30	Multiangle
Ref. [18] (2016, Stortini, University of Venice)	Copper	CS (0.4 M), SA (10 mM)	2, PSD (- 0.25 V)	0.071	0.4	1E8	12.57$	10	Multiangle
Ref. [19] (2016, Roustaie, TU Darmstadt)	Gold	Gold SF	3, PSD (50 ms / - 0.45 V; 50 ms / - 0.9 V)	>3	0.4	1.6E6	0.2$	25	Parallel
Ref. [20] (2017, Barako, Stanford University)	Copper	CS (0.6 M), SA (30 mM)	3, GSD (-0.32 V)	0.25	0.2<br>0.4<br>1	-	-	30	Multiangle
Ref. [21] (2017, Roustaie, TU Darmstadt)	Gold<br>Nickel	Metakem Goldbad SF<br>Ni(SO3NH2)2 (- M), H3BO3 (- M)	-, PED (10 ms / -0.6V; 40ms / - 0.05 V)<br>-, PED (10 ms / - 0.9 V; 40 ms / - 0.45 V)	1	3.5<br>7	2E6	19.24$<br>76.97$	-	Parallel<br>Cones
Ref. [22] (2018, Bieker, TU Darmstadt)	Gold	-	-	-	3<br>3.6<br>3.75	6E4<br>4E5<br>1E6	0.42$<br>4.07$<br>11.04$	24	Parallel<br>Cones
Ref. [23] (2020, Jiang, Loughborough University)	Copper	CS (0.8 M), SA (5 mM$)	2, PSD (0.3V)	-	0.15	-	-	8	Multiangle
Ref. [24–26] (2020-2021, Roustaie, Nanowired, TU Darmstadt)	Copper	-	-	1200#	0.03 – 4#	-	-	50#	Parallel, Multiangle
Ref. [27] (2021, Strahringer, Nanowired)	Copper	-	-	1200#	0.1<br>0.4<br>1	3.8E9$<br>2.4E8$<br>-	30<br>30<br>-	10 - 45	Parallel
Ref. [28] (2023, Qiao, Stanford University)	Copper	CS (0.6 M)	3, PSD (- 0.32 V)	0.25	0.45	-	-	>20	Multiangle
Ref. [29,30] (2024, Bickel, Nanowired)	Copper	-	-	700	0.1<br>0.4	3.8E9$<br>2.4E8$	30<br>30	3 - 6	Parallel
Ref. [31] (2024, Zhu, Temple University)	Copper	CS (0.6 M), SA (1M)	2, PSD (- 0.4V)	1	0.4	1.5E8$	19	20	Multiangle
This work	Copper	CSH (1M), SA(0.25M)	2, PED (40 ms / 0.1V; 40 ms / 0.05V)	12	0.1<br>0.2<br>0.4<br>0.4<br>1	3.8E9<br>6.0E8<br>2.6E8<br>1.5E8<br>2.2E7	29.85<br>18.85<br>32.67<br>18.85<br>17.28	10<br>10<br>10<br>3 - 20<br>10	Parallel, Multiangle

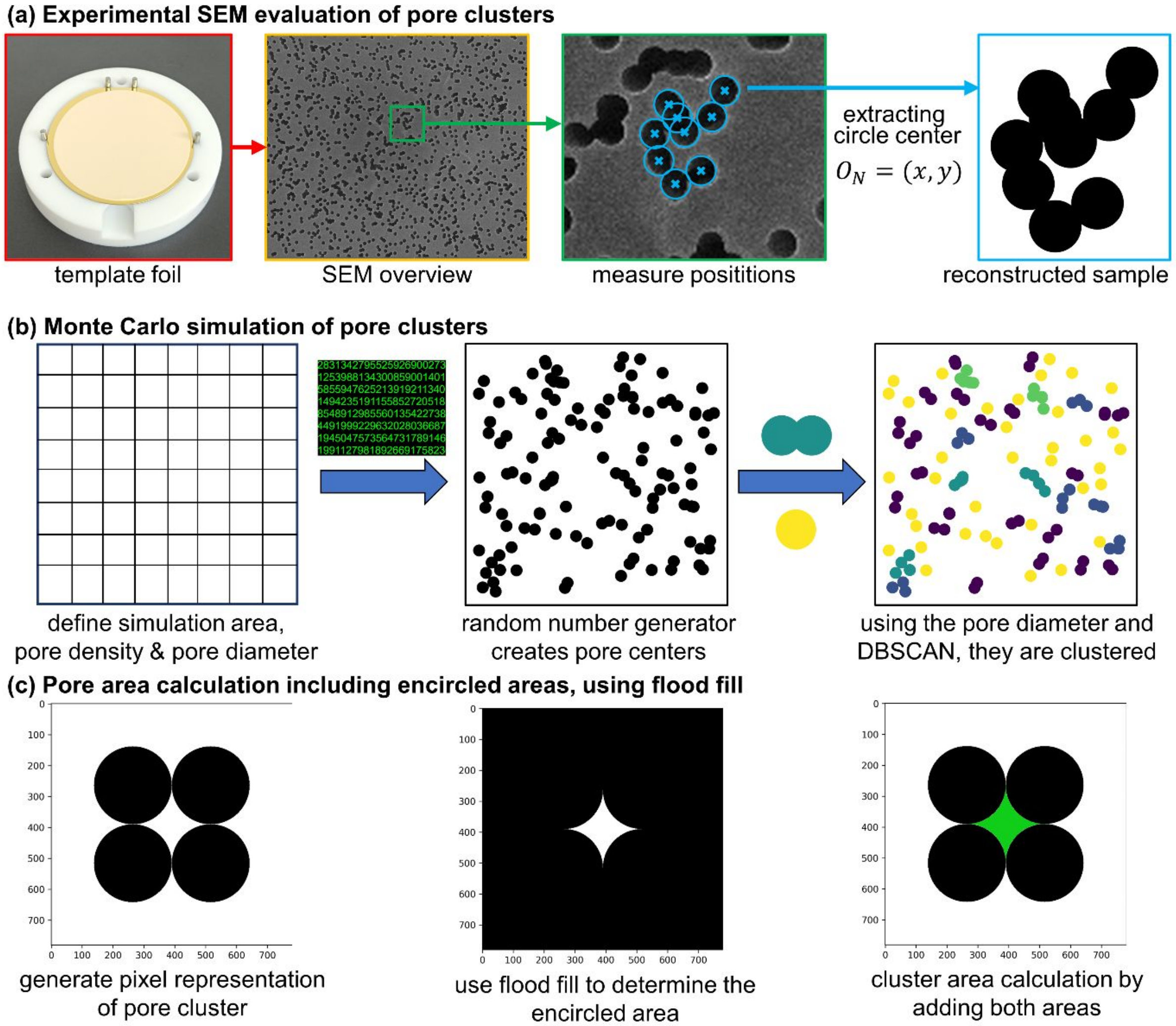

Figure S1: Workflow for experimental template characterization and Monte Carlo simulation. (a) SEM-based experimental characterization of template morphology. The workflow consists of: 1, Sample Preparation: The template is coated with a thin titanium layer using e-beam evaporation. 2, Image Acquisition: Afterwards the template can be analyzed in the SEM, allowing for high magnification captures of the template surface. 3, Image Analysis: Subsequently, the SEM images are analyzed and the pore centers are extracted via imageJ. (b) Monte Carlo (MC) simulation of pore clustering. The simulation workflow consists of: 1, Pore Generation: First, a uniform random number generator is used to assign x, y coordinates to pore centers within the defined simulation area, ensuring a uniform spatial distribution based on the specified pore density. 2, Cluster Identification: A clustering algorithm, DBSCAN, is then applied to identify groups of overlapping pores, where clustering occurs when the distance between adjacent pore centers is smaller than the pore diameter. DBSCAN was specifically chosen for its ability to handle spatial clustering without pre-defined cluster numbers, making it ideal for capturing realistic clustering behavior of randomly distributed pores. (c) 3, Area Calculation: To determine the area of each cluster, we transform the clustered pores into a pixel-based representation and apply a flood fill algorithm to estimate the total covered area. Flood fill was chosen due to the ability to account for encircled areas as they also add area to the etched pore due to being completely detached from the membrane.

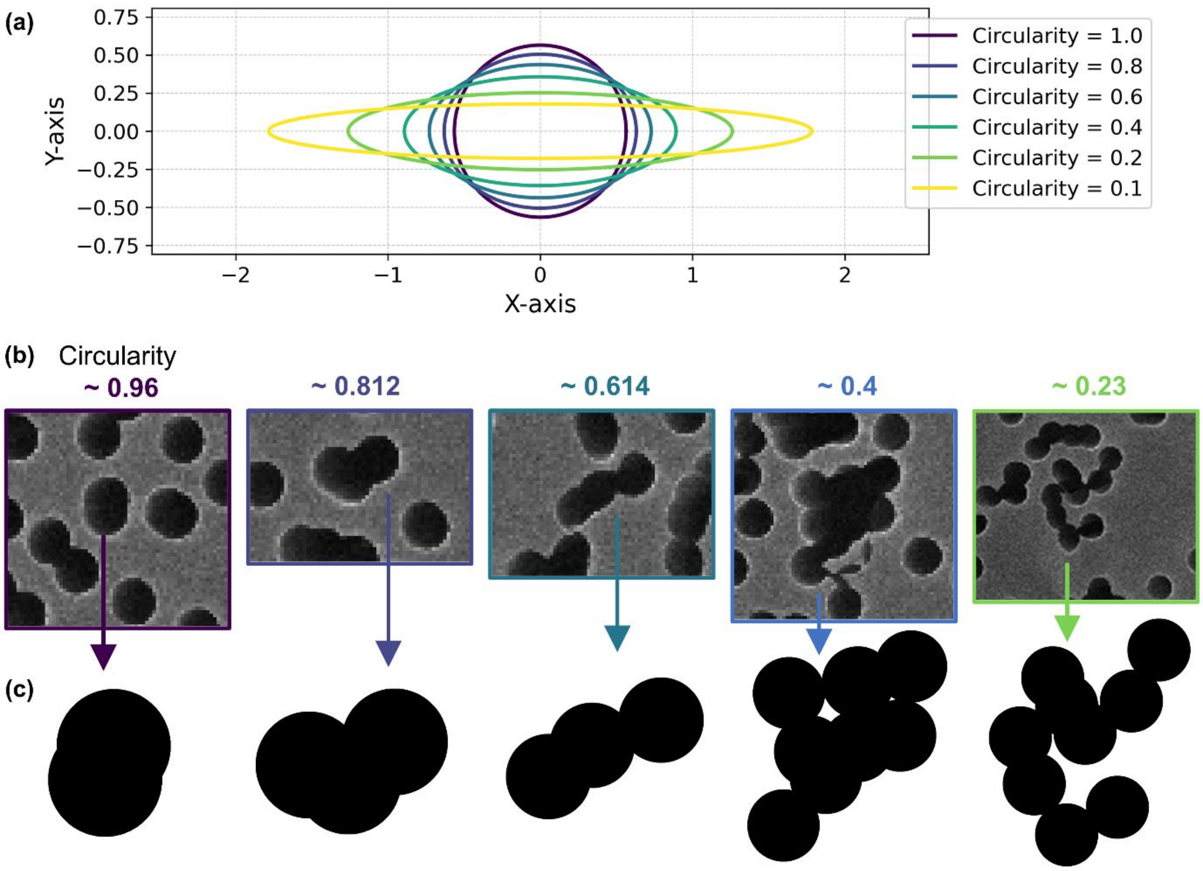

Figure S2: Circularity of experimental and reconstructed pore clusters. (a) A visual reference scale for the circularity metric (calculated as 4π * Area / Perimeter²). A series of ellipses with decreasing circularity values, from a perfect circle (1.0) to a highly elongated shape (0.1). (b) Examples of experimentally observed multipore clusters from SEM images of the templates, each labeled with its calculated circularity value. (c) Digital reconstructions of the corresponding clusters from panel (b), generated from the identified pore center coordinates.

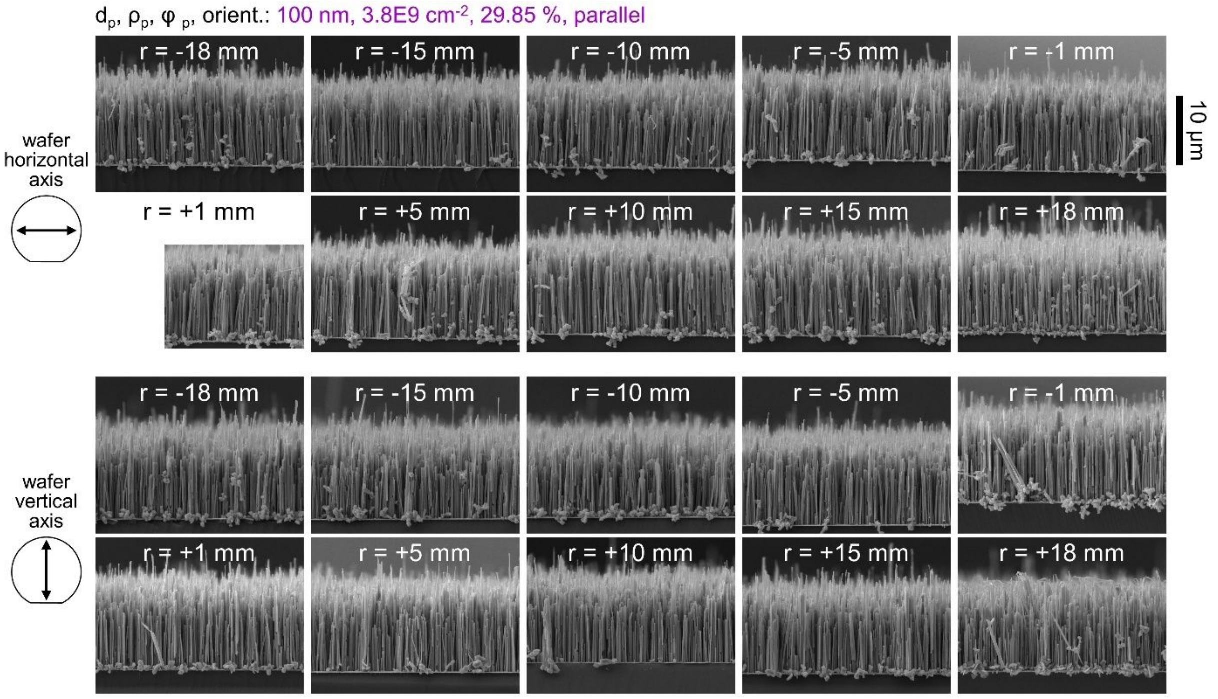

Figure S3: SEM cross sectional analysis to reveal length of OSE copper nanowires at various radial distances. Sample prepared by template T1.

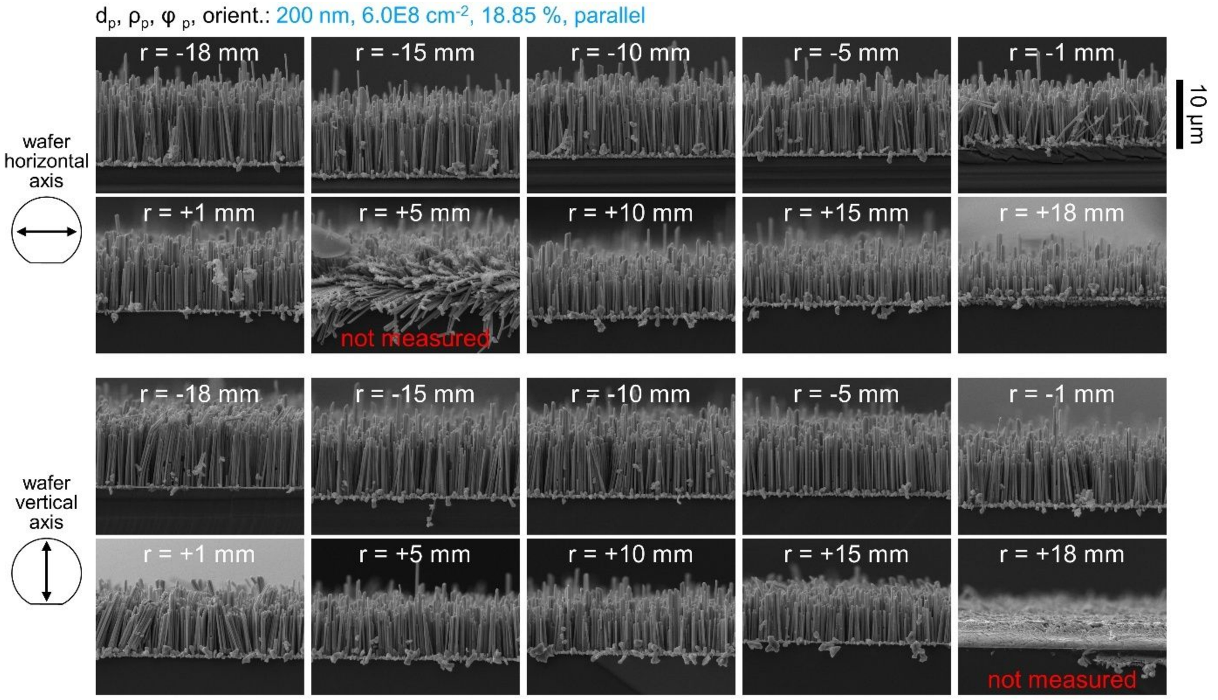

Figure S4: SEM cross sectional analysis to reveal length of OSE copper nanowires at various radial distances. Sample prepared by template T2.

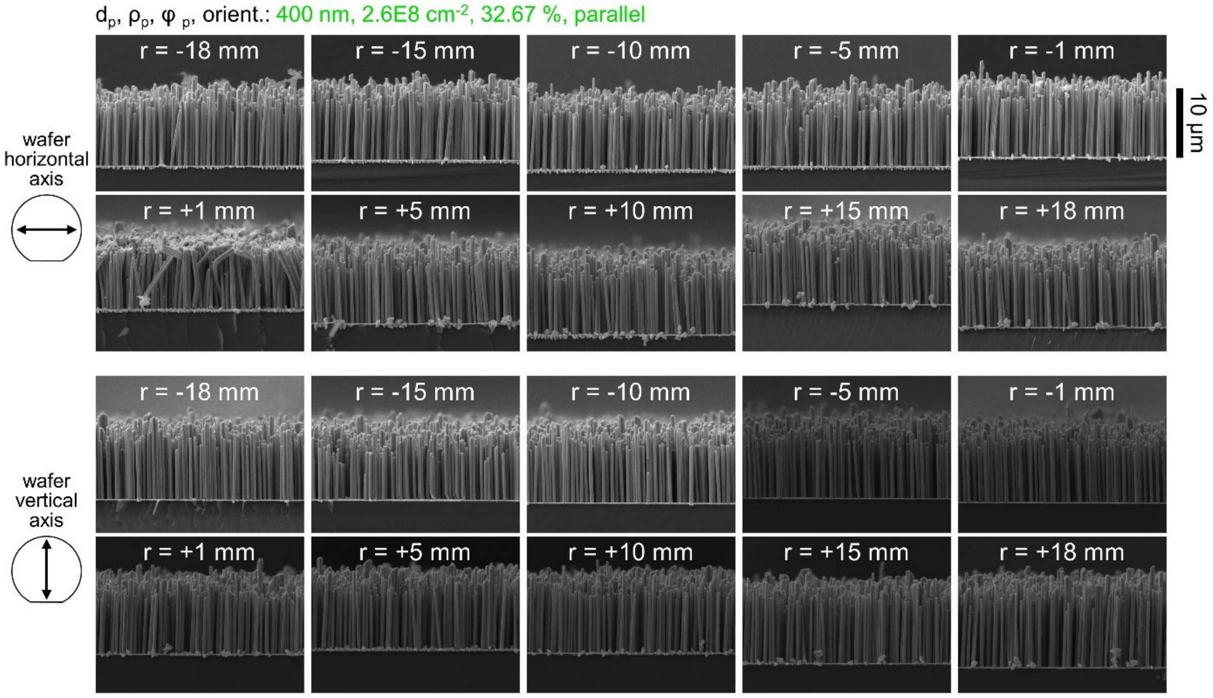

Figure S5: SEM cross sectional analysis to reveal length of OSE copper nanowires at various radial distances. Sample prepared by template T3.

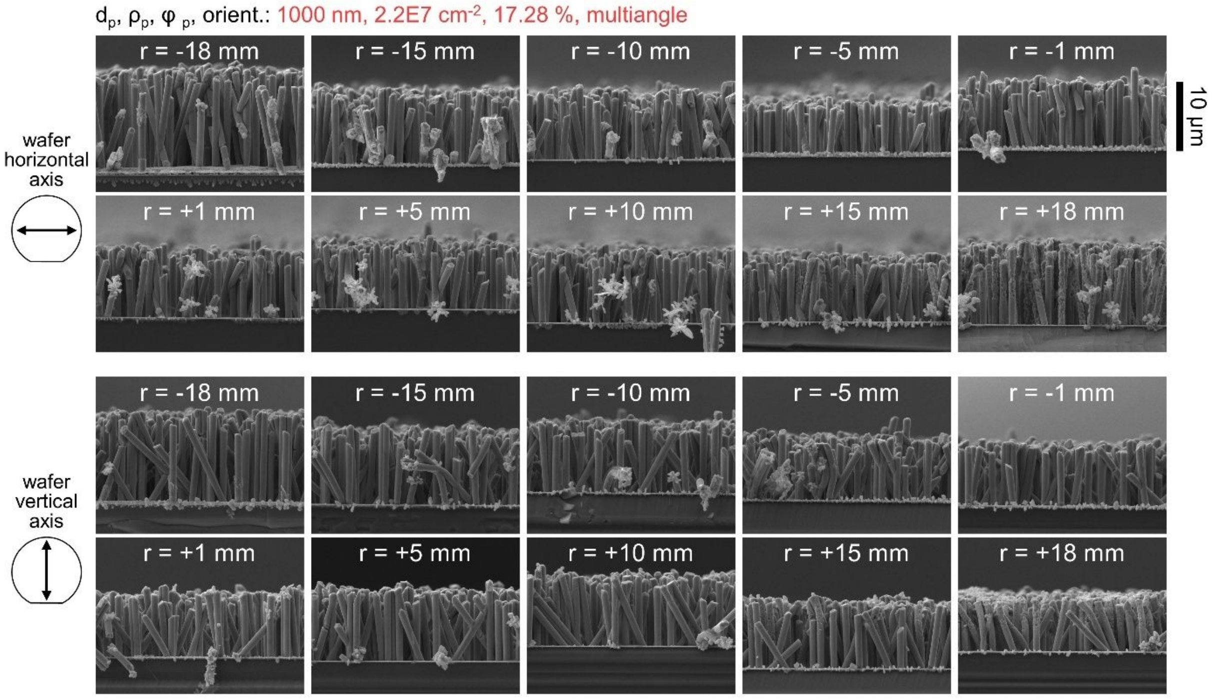

Figure S6: SEM cross sectional analysis to reveal length of OSE copper nanowires at various radial distances. Sample prepared by template T5.

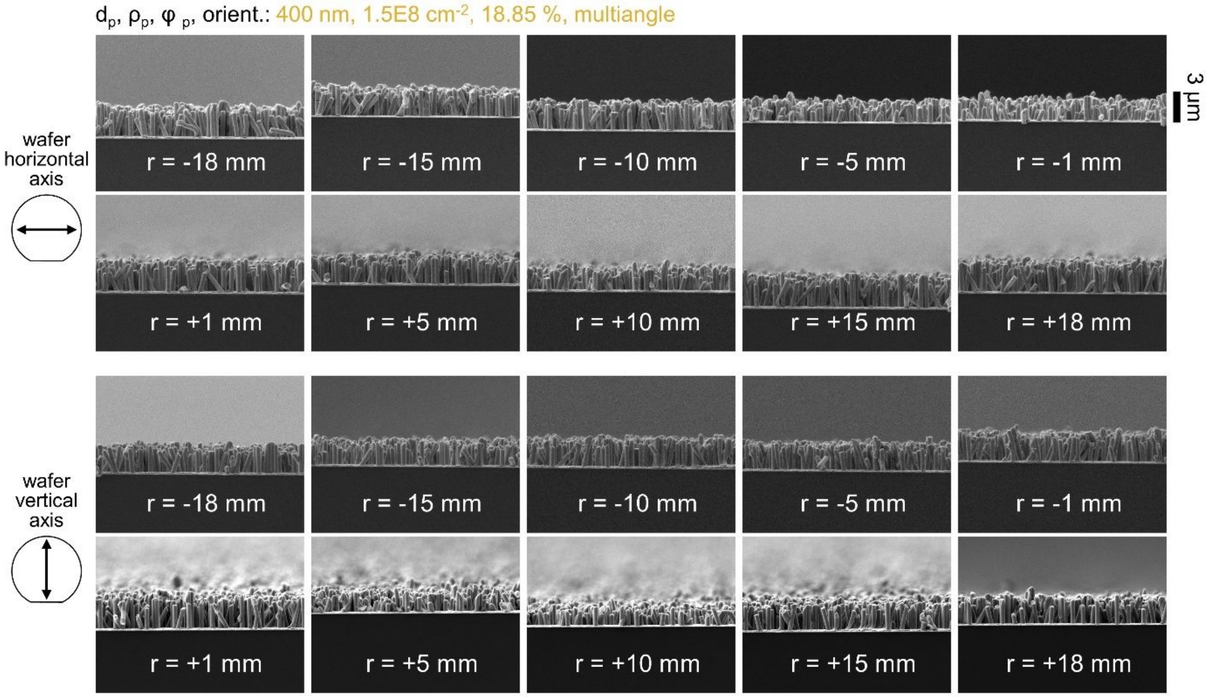

Figure S7: SEM cross sectional analysis to reveal length of OSE copper nanowires at various radial distances. Sample prepared by template T4 and plating charge $q_{plating}$ = 18.50 As.

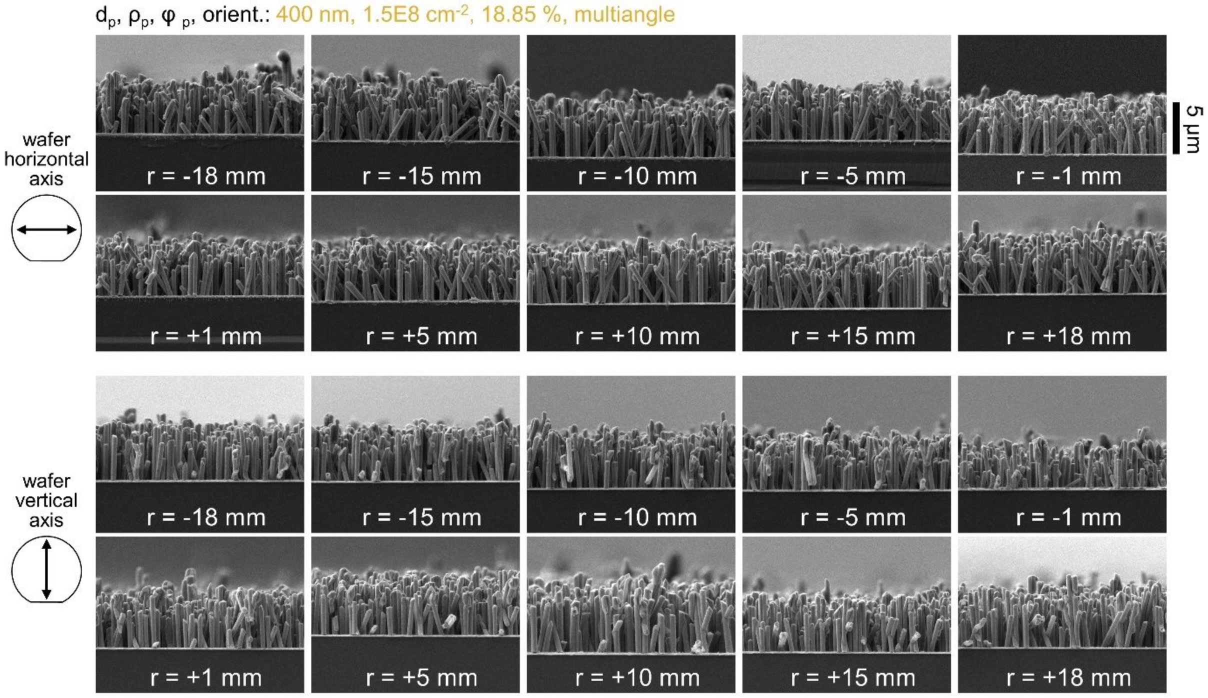

Figure S8 SEM cross sectional analysis to reveal length of OSE copper nanowires at various radial distances. Sample prepared by template T4 and plating charge $q_{plating}$ = 30.86 As.

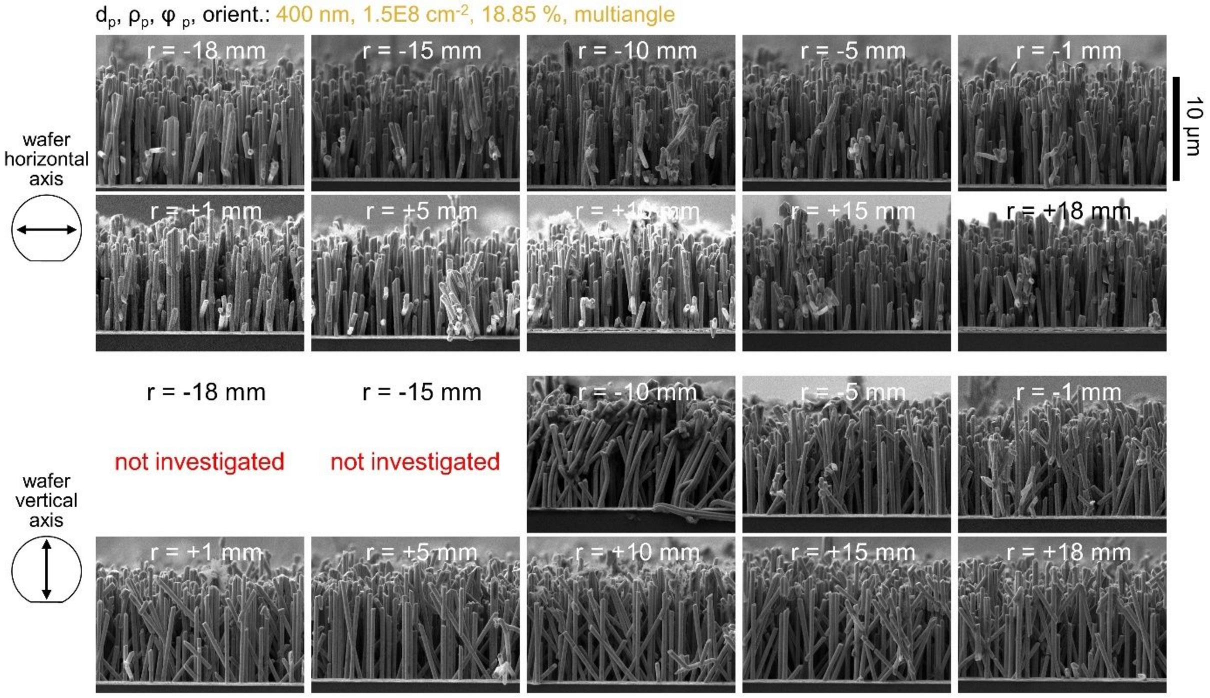

Figure S9: SEM cross sectional analysis to reveal length of OSE copper nanowires at various radial distances. Sample prepared by template T4 and plating charge $q_{plating}$ = 60.80 As.

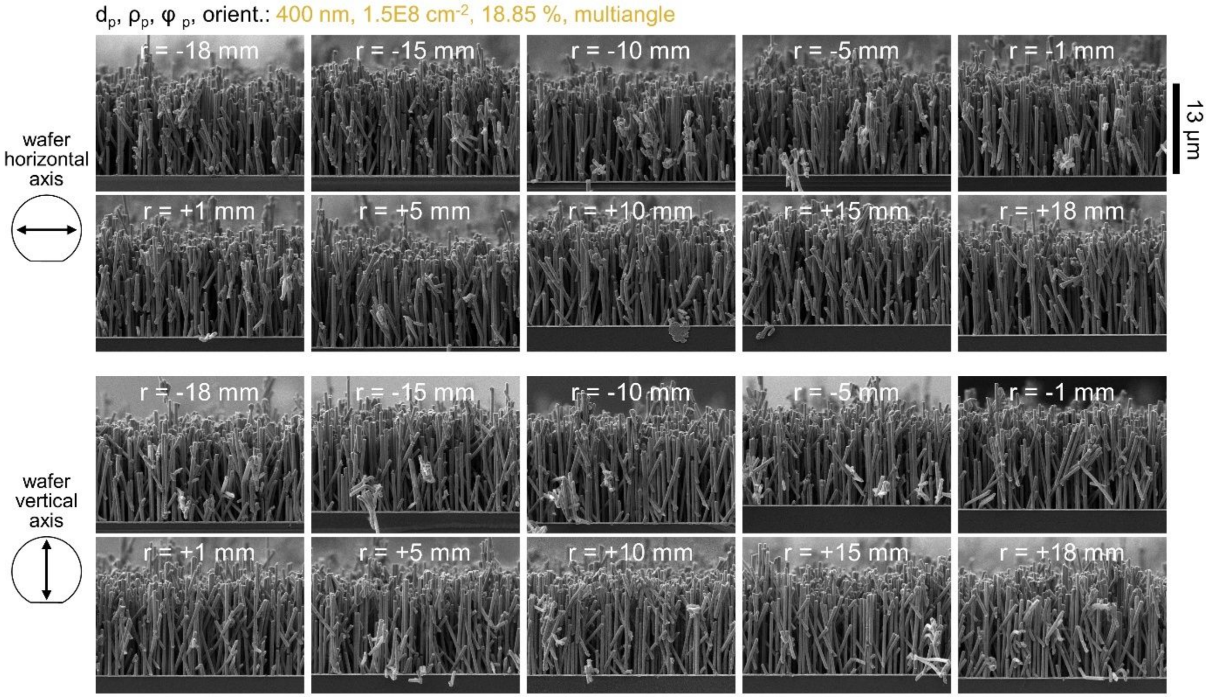

Figure S10: SEM cross sectional analysis to reveal length of OSE copper nanowires at various radial distances. Sample prepared by template T4 and plating charge $q_{plating}$ = 92.51 As.

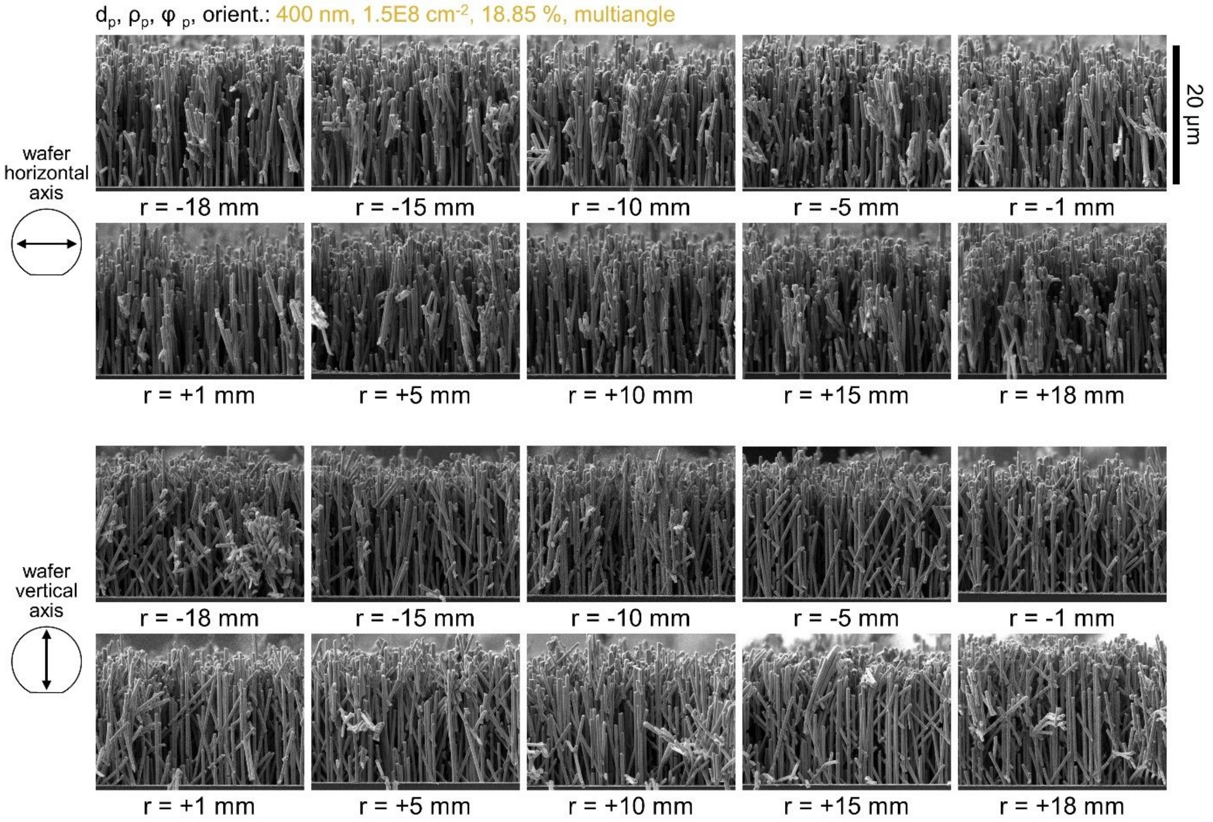

Figure S11: SEM cross sectional analysis to reveal length of OSE copper nanowires at various radial distances. Sample prepared by template T4 and plating charge $q_{plating}$ = 120 As.

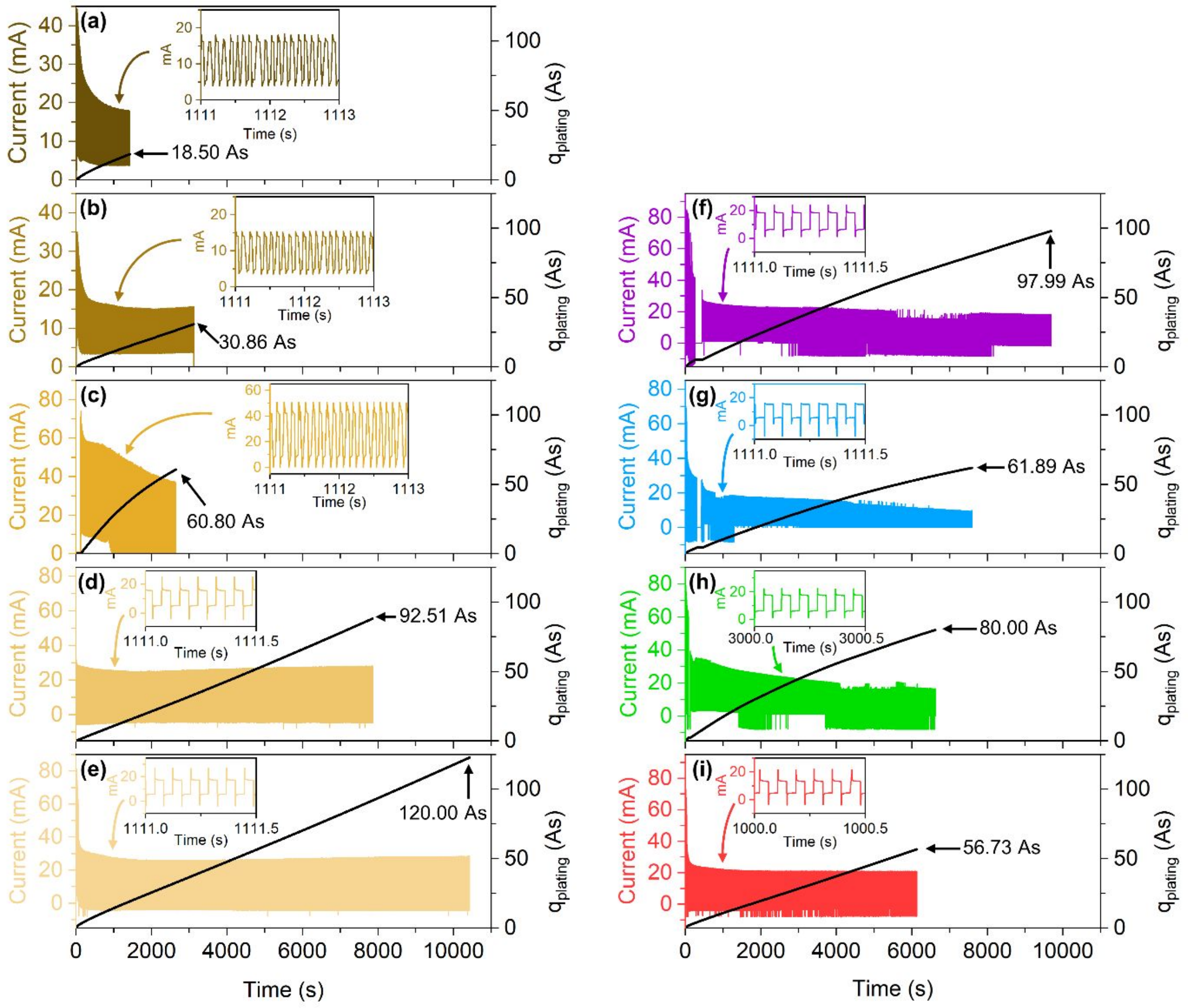

Figure S12: In potentiostatic template-assisted electrodeposition, chronoamperograms (left colored axes) help monitoring the evolution of copper metal nanowires. Panels **(a-e)** refer to fabrication of OSE-made copper nanowires with tailored length based on template T4. Panels **(f, g, h, i)** are obtained during sample preparation based on template T1, T2, T3 and T5, respectively. Each sample is obtained with a pulsed-electrodeposition mode by applying alternating potentiostatic pulses of 0.1 V and 0.05 V for 40 ms each. The cumulative plating charge $q_{plating}$ (right black axis) is the essential control parameter for tuning the length of the wires. The length of the as-grown metal nanowires results from the final value of $q_{plating}$ (indicated by black arrows) in conjunction with the template characteristics (i.e., pore diameter, density or porosity), growth area (i.e., defined by diameter of plating chamber), and the Faradaic efficiency of the process (i.e., in case of copper sulfate electrolyte solution with pH 1 deposited at a range of 0.05 V to 0.1 V leads to an efficiency of about unity with no parasitic side reactions).

References

(1) Riedel, C.; Spohr, R. Statistical Properties of Etched Nuclear Tracks: I. Analytical Theory and Computer Simulation. *Radiat. Eff.* **1979**, *42* (1–2), 69–75. https://doi.org/10.1080/10420157908201738.

(2) Riedel, C.; Spohr, R. Statistical Properties of Etched Nuclear Tracks II. Experiment and Filter Design. *Radiat. Eff.* **1980**, *46* (1–2), 23–29. https://doi.org/10.1080/00337578008209148.

(3) Riedel, C.; Spohr, R. Correcting Overlapping Counts in Dose Calibration at High Event-Densities. *Nucl. Tracks* **1981**, *5* (3), 265–270. https://doi.org/10.1016/0191-278X(81)90004-4.

(4) Kim, K.-J.; Stevens, P. V.; Fane, A. G. Porosity Dependence of Pore Entry Shape in Track-Etched Membranes by Image Analysis. *J. Membr. Sci.* **1994**, *93* (1), 79–90. https://doi.org/10.1016/0376-7388(94)85018-6.

(5) Calvo, J. I.; Hernández, A.; Caruana, G.; Martı́nez, L. Pore Size Distributions in Microporous Membranes: I. Surface Study of Track-Etched Filters by Image Analysis. *Journal of Colloid and Interface Science* **1995**, *175* (1), 138–150. https://doi.org/10.1006/jcis.1995.1439.

(6) Shorin, V. S. A Statistical Model for Hole Distribution on the Nuclear-Track Membrane Surface. *High Energy Chem.* **2001**, *35* (4), 229–235. https://doi.org/10.1023/A:1017680427207.

(7) Shorin, V. S. Hole Overlapping Statistics in Reactor Nuclear Track Membranes. *High Energy Chem.* **2003**, *37* (3), 162–168. https://doi.org/10.1023/A:1024147202920.

(8) Sawada, S. Computer Simulation to Predict Size Distribution of Track-Etched Nanopores. *Jpn. J. Appl. Phys.* **2022**, *61* (4), 046005. https://doi.org/10.35848/1347-4065/ac4b6d.

(9) Jianqiang, F. U. Hole Distribution on the Nuclear Track-Etched Membrane Surface. *zggx* **2024**, *45* (9), 90201. https://doi.org/10.11889/j.0253-3219.2022.hjs.45.090201.

(10) Vila, L.; Vincent, P.; Dauginet-De Pra, L.; Pirio, G.; Minoux, E.; Gangloff, L.; Demoustier-Champagne, S.; Sarazin, N.; Ferain, E.; Legras, R.; Piraux, L.; Legagneux, P. Growth and Field-Emission Properties of Vertically Aligned Cobalt Nanowire Arrays. *Nano Lett.* **2004**, *4* (3), 521–524. https://doi.org/10.1021/nl0499239.

(11) Yoon, H.; Deshpande, D. C.; Ramachandran, V.; Varadan, V. K. Aligned Nanowire Growth Using Lithography-Assisted Bonding of a Polycarbonate Template for Neural Probe Electrodes. *Nanotechnology* **2007**, *19* (2), 025304. https://doi.org/10.1088/0957-4484/19/02/025304.

(12) Gambirasi, A.; Cattarin, S.; Musiani, M.; Vázquez-Gómez, L.; Verlato, E. Direct Electrodeposition of Metal Nanowires on Electrode Surface. *Electrochim. Acta* **2011**, *56* (24), 8582–8588. https://doi.org/10.1016/j.electacta.2011.07.045.

(13) Cui, Y.; Ju, Y.; Wang, P.; Xu, B.; Kojima, N.; Ichioka, K.; Hosoi, A. Carbon Nanotube–Cu/Parylene Nanowire Array Electrical Fasteners with High Adhesion Strength. *Appl. Phys. Express* **2013**, *7* (1), 015102. https://doi.org/10.7567/APEX.7.015102.

(14) Greiner, F.; Quednau, S.; Dassinger, F.; Sarwar, R.; Schlaak, H. F.; Guttmann, M.; Meyer, P. Fabrication Techniques for Multiscale 3D-MEMS with Vertical Metal Micro- and Nanowire Integration. *J. Micromech. Microeng.* **2013**, *23* (2), 025018. https://doi.org/10.1088/0960-1317/23/2/025018.

(15) Wang, P.; Ju, Y.; Cui, Y.; Hosoi, A. Copper/Parylene Core/Shell Nanowire Surface Fastener Used for Room-Temperature Electrical Bonding. *Langmuir* **2013**, *29* (45), 13909–13916. https://doi.org/10.1021/la402475f.

(16) Motoyama, M.; Prinz, F. B. Electrodeposition and Behavior of Single Metal Nanowire Probes. *ACS Nano* **2014**, *8* (4), 3556–3566. https://doi.org/10.1021/nn4066582.

(17) Barako, M. T.; Roy-Panzer, S.; English, T. S.; Kodama, T.; Asheghi, M.; Kenny, T. W.; Goodson, K. E. Thermal Conduction in Vertically Aligned Copper Nanowire Arrays and Composites. *ACS Appl. Mater. Interfaces* **2015**, *7* (34), 19251–19259. https://doi.org/10.1021/acsami.5b05147.

(18) Stortini, A. M.; Moretto, L. M.; Mardegan, A.; Ongaro, M.; Ugo, P. Arrays of Copper Nanowire Electrodes: Preparation, Characterization and Application as Nitrate Sensor. *Sens. Actuators, B* **2015**, *207*, 186–192. https://doi.org/10.1016/j.snb.2014.09.109.

(19) Roustaie, F.; Quednau, S.; Dassinger, F.; Schlaak, H. F.; Lotz, M.; Wilfert, S. In Situ Synthesis of Metallic Nanowire Arrays for Ionization Gauge Electron Sources. *J. Vac. Sci. Technol. B* **2016**, *34* (2), 02G103. https://doi.org/10.1116/1.4939756.
(20) Barako, M. T.; Isaacson, S. G.; Lian, F.; Pop, E.; Dauskardt, R. H.; Goodson, K. E.; Tice, J. Dense Vertically Aligned Copper Nanowire Composites as High Performance Thermal Interface Materials. *ACS Appl. Mater. Interfaces* **2017**, *9* (48), 42067–42074. https://doi.org/10.1021/acsami.7b12313.
(21) Roustaie, F.; Bieker, J.; Cicek, R.; Schlaak, H. F. Novel Fabrication Method for Integration of Template Grown Metallic Nanocones with Controllable Tip Diameter and Apex Angle. *Microelectron. Eng.* **2017**, *180*, 81–85. https://doi.org/10.1016/j.mee.2017.06.003.
(22) Bieker, J.; Roustaie, F.; Schlaak, H. F.; Langer, C.; Schreiner, R.; Lotz, M.; Wilfert, S. Field Emission Characterization of *in Situ* Deposited Gold Nanocones with Variable Cone Densities. *J. Vac. Sci. Technol., B:Nanotechnol. Microelectron.:Mater., Process., Meas., Phenom.* **2018**, *36* (2), 02C105. https://doi.org/10.1116/1.5009504.
(23) Jiang, H.; Robertson, S.; Zhou, Z.; Liu, C. Cu-Cu Bonding with Cu Nanowire Arrays for Electronics Integration. In *2020 IEEE 8th Electronics System-Integration Technology Conference (ESTC)*; 2020; pp 1–6. https://doi.org/10.1109/ESTC48849.2020.9229670.
(24) ROUSTAIE, F.; QUEDNAU, S.; DASSINGER, F.; BIRLEM, O. Room Temperature Interconnection Technology for Bonding Fine Pitch Bumps Using NanoWiring, KlettWelding, KlettSintering and KlettGlueing. In *2020 15th International Microsystems, Packaging, Assembly and Circuits Technology Conference (IMPACT)*; 2020; pp 168–171. https://doi.org/10.1109/IMPACT50485.2020.9268570.
(25) Roustaie, F.; Quednau, S.; Weißenborn, F.; Birlem, O. Low-Resistance Room-Temperature Interconnection Technique for Bonding Fine Pitch Bumps. *J. Mater. Eng. Perform.* **2021**, *30* (5), 3173–3177. https://doi.org/10.1007/s11665-021-05649-9.
(26) Roustaie, F.; Quednau, S.; Weissenborn, F.; Birlem, O.; Riehl, D.; Ding, X.; Kramer, A.; Hofmann, K. Room Temperature KlettWelding Interconnect Technology for High Performance CMOS Logic. In *2021 IEEE 71st Electronic Components and Technology Conference (ECTC)*; 2021; pp 371–376. https://doi.org/10.1109/ECTC32696.2021.00069.
(27) Strahringer, D.; Roustaie, F.; Weissenborn, F.; Quednau, S.; Wilde, J. Optimizing the Nano Wiring and KlettSintering Parameters for Low-Temperature Die to DCB Attach of Power Electronic Chips. In *2021 16th International Microsystems, Packaging, Assembly and Circuits Technology Conference (IMPACT)*; 2021; pp 31–34. https://doi.org/10.1109/IMPACT53160.2021.9696635.
(28) Qiao, H.; Jiang, K.; Wei, T.; Lin, Y.; Perez, C.; Asheghi, M.; Goodson, K. Development of Thermal Interface Materials Tape Using Vertically Aligned Copper Nanowire-PDMS Composites. In *ASME 2023 International Technical Conference and Exhibition on Packaging and Integration of Electronic and Photonic Microsystems*; American Society of Mechanical Engineers: San Diego, California, USA, 2023; p V001T07A007. https://doi.org/10.1115/IPACK2023-113126.
(29) Bickel, S.; Quednau, S.; Birlem, O.; Panchenko, J.; Junghähnel, M. Cu Nanowire Fine-Pitch Joints for next Gen Heterogeneous Chiplet Integration. In *2024 IEEE 74th Electronic Components and Technology Conference (ECTC)*; IEEE: Denver, CO, USA, 2024; pp 1376–1381. https://doi.org/10.1109/ECTC51529.2024.00224.
(30) Bickel, S.; Quednau, S.; Birlem, O.; Graff, A.; Altmann, F.; Junghähnel, M.; Panchenko, J. Fine-Pitch Copper Nanowire Interconnects for 2.5/3D System Integration. *J. Electron. Mater.* **2024**. https://doi.org/10.1007/s11664-024-11107-8.
(31) Zhu, L.; Li, T.; Wang, H.; Kumara, C.; Gao, W.; Ren, F. Fabrication and Performance Evaluation of Double-Sided Copper Nanowire Arrays as Thermal and Electrical Interfacial Layers. *ACS Appl. Electron. Mater.* **2024**, acsaelm.4c00797. https://doi.org/10.1021/acsaelm.4c00797.
